# A genetic biosensor for identification of transcriptional repressors of target promoters

**DOI:** 10.1038/srep15887

**Published:** 2015-10-29

**Authors:** Weishan Wang, Xiao Li, Yue Li, Shanshan Li, Keqiang Fan, Keqian Yang

**Affiliations:** 1 State Key Laboratory of Microbial Resources, Institute of Microbiology, Chinese Academy of Sciences, Beijing, 100101, People’s Republic of China

## Abstract

Transcriptional repressors provide widespread biological significance in the regulation of gene expression. However, in prokaryotes, it is particularly difficult to find transcriptional repressors that recognize specific target promoters on genome-scale. To address this need, a genetic biosensor for identifying repressors of target promoters was developed in *Escherichia coli* from a *de novo* designed genetic circuit. This circuit can convert the negative input of repressors into positive output of reporters, thereby facilitating the selection and identification of repressors. After evaluating the sensitivity and bias, the biosensor was used to identify the repressors of *scbA* and *aco* promoters (P*scbA* and P*aco*), which control the transcription of signalling molecule synthase genes in *Streptomyces coelicolor* and *Streptomyces avermitilis,* respectively. Two previously unknown repressors of P*scbA* were identified from a library of TetR family regulators in *S. coelicolor*, and three novel repressors of P*aco* were identified from a genomic library of *S. avermitilis*. Further *in vivo* and *in vitro* experiments confirmed that these newly identified repressors attenuated the transcription of their target promoters by direct binding. Overall, the genetic biosensor developed here presents an innovative and powerful strategy that could be applied for identifying genome-wide unknown repressors of promoters in bacteria.

Gene expression in prokaryotes is predominantly regulated at the transcriptional level^1^. Promoter regions of key genes, such as the biofilm gene (*csgD*) in *Escherichia coli*^2^, are subjected to complex transcription regulation. Hence, the on and off status of key genes are fine-tuned by numerous transcription factors (TFs), including repressors, activators and sigma factors^3^. These TFs usually form sophisticated regulatory networks to temporally control the expression patterns of key genes via dynamic interactions with target promoters in response to growth and environmental signals^1^,^3^.

To dissect the interactions in transcriptional regulatory networks, several powerful methods for the determination of the DNA-binding sites of TFs, such as SELEX^4^, ChIP-chip^5^, ChIP-seq^6^,^7^ and the bacterial one-hybrid system^8^, have been developed. However, in the reverse direction, strategies to identify TFs of target promoters remain limited. Currently available techniques to identify TFs binding to specific promoters include a modified bacterial one-hybrid reporter system^9^ and the DNA capture strategies^10^,^11^,^12^. The modified bacterial one-hybrid reporter system requires the fused expression of the DNA binding domain of candidate TFs with the α subunit of RNA polymerase in *E. coli*. Theoretically, if the recombinant TFs could interact with the target DNA, they would recruit and stabilize the binding of RNA polymerase at the core promoter region and thus enhance the transcription of reporter genes^9^. The potential challenge of this strategy implies that the fused TF expression libraries may not be designed efficiently, and as a result the fused TFs may be nonfunctional^9^. The *in vitro* DNA capture strategies by DNA pull-down^13^,^14^,^15^ or DNA affinity chromatography capture^10^,^11^ enable the enrichment of binding TFs from a cell-free extract. Subsequently, the captured TFs are identified by mass spectrometry. This strategy therefore is limited by possible under-presentation of certain TFs, such as those not expressed or expressed at low levels at a given sampling time^10^,^11^,^12^,^13^,^14^,^15^. In addition, the abovementioned methods cannot distinguish between activators and repressors. Therefore, to untangle the transcriptional regulatory networks, efficient and convenient methods for identifying regulators of target promoters still need to be developed.

Transcriptional repressors and activators are important components of a transcription network^3^. From the view of signal processing, TFs can be considered as input, whereas the transcripts controlled by their target promoter represent the outputs. Given that activators exert positive effects on the output, identification of the activators of a target gene or operon is relatively straightforward; in fact, large-scale screening methods were developed to identify activators^16^,^17^. However, using the attenuated output signals is difficult to identify repressors. Recently, various genetic circuits have been constructed and utilised for different applications^18^. We hypothesised that a NOT gate (the output is OFF when the input is ON and vice versa) genetic circuit^19^ can be built to perform the signal-processing function of converting negative input into positive output, and such a circuit could be developed as a biosensor to detect the repressors of target promoters.

Streptomycetes are the most abundant source of bioactive secondary metabolites, such as anti-fungals, anti-virals, anti-tumorals, anti-hypertensives, immunosuppressants, and especially antibiotics^20^. In streptomycetes, the signalling molecules γ-butyrolactones (GBLs) and their cognate receptors are widely used to regulate the onset of secondary metabolism and morphological differentiation^21^. In *Streptomyces coelicolor*, the major GBL molecule is SCB1^21^, which is synthesized by ScbA^22^. The transcription of *scbA* is temporally controlled and only switched on at the transition period^22^,^23^. In *Streptomyces avermitilis*, the producer of the important antibiotic avermectin, another butenolide-type signalling molecule avenolide is essential for eliciting avermectin production^24^. Similar to SCB1, the temporal production of avenolide is intricately related to the growth physiology of *S. avermitilis*^24^. Therefore, to gain deeper understanding of the control of signalling molecule production in different *Streptomyces*, unknown repressors recognising the promoter regions of these signalling molecule synthase genes should be identified.

In this work, a genetic biosensor which can convert the negative input of repressors into the positive output of reporters was *de novo* constructed in *E. coli*. The promoters of *scbA* and *aco* (P*scbA* and P*aco*) were selected as baits to fish out unknown repressors in *S. coelicolor* and *S*. *avermitilis*, respectively. To easily select and conveniently quantify the binding of repressors with these target promoters, *xylE-neo* cassette was built as the output reporter in this biosensor^25^. This genetic biosensor was first evaluated by selecting the known repressors of a promoter (P*kasO*) from *S. coelicolor*. Then it was applied in the selection of repressors of P*scbA* and P*aco* from a repressor library and a genomic DNA library, respectively. Two new repressors of P*scbA* and three novel repressors of P*aco* were identified. Overall, this study demonstrates that the genetic biosensor-based strategy can be instrumental in the discovery of new repressors of target promoters, and thereby providing new insights into the complex regulation of key promoter regions in bacteria.

## Results

### Design of the genetic biosensor for detecting repressors of target promoters

**Figure 1:**
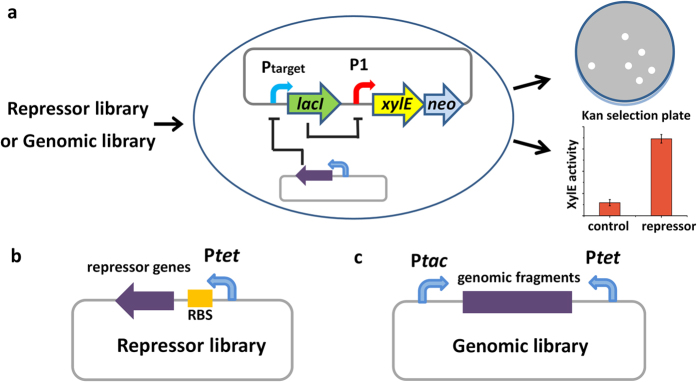
Schematic illustration of the genetic biosensor. (**a**) Principle of the genetic biosensor. Promoter of interest (Ptarget) is cloned into the genetic circuit plasmid pCSW3. (**b**) Design of the repressor library. Candidate repressor genes are cloned into pACW1. (**c**) Design of the genomic library. Random genomic fragments are cloned into pACW2.

To identify unknown repressors of target promoters on genome-wide scale, we designed a genetic biosensor to convert the negative input of repressors into the positive output of reporters (Fig. 1a). In the biosensor, the sensor-plasmid contains a NOT gate genetic circuit. The circuit was *de novo* constructed with two components: one contains a target promoter controlled *lacI* gene, which responds to the repressors to downregulate the expression of LacI, and the other contains the LacI repressible promoter (P1) controlled reporters, which can react to the level of LacI (Fig. 1a). To facilitate the selection and quantification of the positive output, the double reporter cassette *xylE-neo* was used (Fig. 1a)^25^,^26^. It allows simultaneous detection of two outputs: (1) catechol-2,3-dioxygenase (XylE) catalyses the conversion of colourless catechol into the intensely yellow hydroxymuconic semialdehyde, whose activity can be measured in colorimetric assays or observed on plate; (2) kanamycin resistance (Neo, aminoglycoside 3′-phosphotransferase) allows the efficient selection of positive clones on plates containing kanamycin.

Two approaches were designed to construct the libraries for selection (Fig. 1b,c). To construct repressor library, the plasmid pACW1 was used. The candidate repressor genes are inserted downstream of the promoter of tetracycline resistance gene (P*tet*) with an appropriate ribosome binding site (RBS) ((Supplementary Fig. S1a). To construct genomic library, the plasmid pACW2 was designed (Supplementary Fig. S1b), which contains two promoters at both sides of the cloning site to improve the expression probability of the coding genes in the inserted DNA fragments. In addition, two XcmI sites were designed at the cloning site to linearise pACW2 and generate a single 3′-overhang thymine residue at both ends, which will facilitate the insertion of DNA fragments by AT cloning.

### Development of LacI-responsive promoters for the output controller component

To select an appropriate promoter that could respond sensitively to the LacI concentration, P*tac* promoter widely used in genetic circuit construction was considered^27^,^28^ (Supplementary Fig. S2a). To evaluate the response of P*tac* to LacI, the plasmid pDR4-P*tac* containing P*tac* driven *xylE-neo* cassette was transformed into *E. coli* JM109, which can express LacI protein constitutively. Although the repression effect of P*tac* by LacI was observed, a high level of leaky expression of the reporter genes was also detected (Supplementary Fig. S2b). To reduce the leaky expression from P*tac*, the replication origin of pDR4-P*tac* was replaced by a low copy number replication origin from pSC101^29^. However, the leaky expression of reporter genes remained very strong (Supplementary Fig. S2b,c). These unexpected results could be attributed to the fact that P*tac* is a strong promoter and its leaky expression is still considerable at the repressed state of LacI in *E. coli*, which clearly suggests that P*tac* does not suitably and sensitively respond to the LacI level.

**Figure 2:**
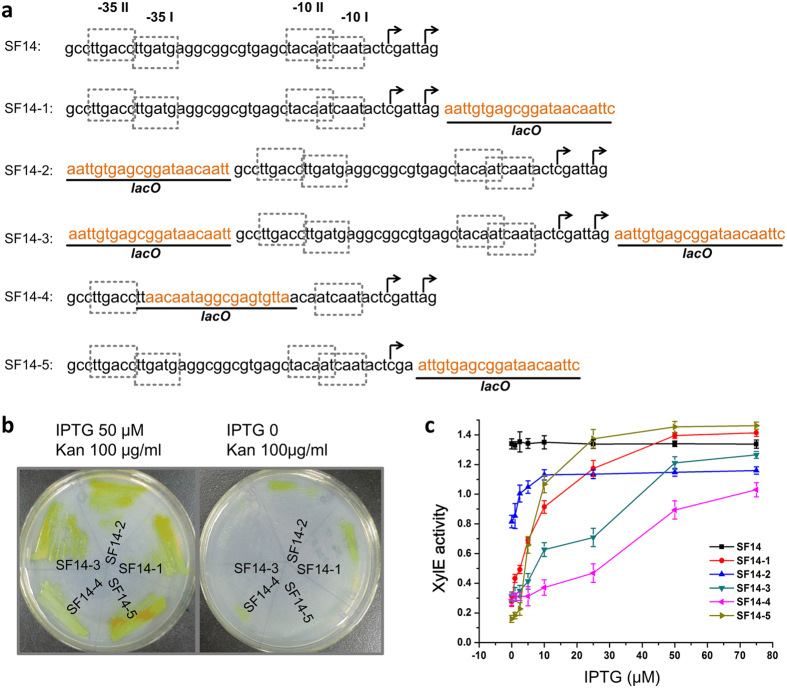
Construction and evaluation of SF14 and *lacO* fusion promoters. (**a**) Sequences of SF14 and *lacO* fusion promoters. The putative transcription start sites are indicated by a bent arrow above the corresponding nucleotide. Sequences of *lacO* are underlined and coloured in light organge. The −10 and −35 regions are marked by dotted boxes. (**b**) Evaluation of SF14 and *lacO* fusion promoters on plates. After incubating the corresponding strains at 37 ^o^C for 12 h, the plates were sprayed with catechol. (**c**) Dynamic expression profiles of SF14 and *lacO* fusion promoters measured by XylE activity induced with different IPTG concentrations. The values are presented as mean ± SD of three independent experiments.

To obtain a promoter more sensitive to the LacI concentration, we decided to develop hybrid promoters using core promoter elements with appropriate activity. The promoter SF14 from *Streptomyces ghanaensis* phage I19^30^, previously demonstrated moderate transcription in *E. coli*^26^, was tested for this purpose. When placed in front of *xylE-neo* reporter cassette, SF14 conferred kanamycin resistance in JM109 (pCSW2-SF14) at approximately 100 μg/ml, which was much lower than the level conferred by P*tac*. Therefore, SF14 was used as a basic component to construct synthetic promoters containing the *lacO* operator. Five different synthetic promoters were assembled by inserting the *lacO* operator at different positions relative to SF14 (Fig. 2a). The reporter activity in *E. coli* JM109 was measured to estimate the performance of these promoters. As expected, both XylE activity (yellow colour) and kanamycin resistance were inhibited on the plates without IPTG induction, indicating LacI in JM109 effectively repressed these synthetic promoters (Fig. 2b). Moreover, these phenotypes were rescued when IPTG was added, indicating that the synthetic promoters respond sensitively to the LacI levels (Fig. 2b). To further characterize the dynamic behaviour of these promoters, we measured XylE activity at different IPTG concentrations (Fig. 2c). Compared with other hybrid promoters, SF14–5 gave the most favourable response curve to the increasing concentration of IPTG: the reported XylE presented the lowest level of leaky expression and the strongest induced expression. Consequently, we chose SF14–5 as the LacI repressible promoter for the control of reporter cassette.

### Construction of the NOT gate genetic circuit

**Figure 3:**
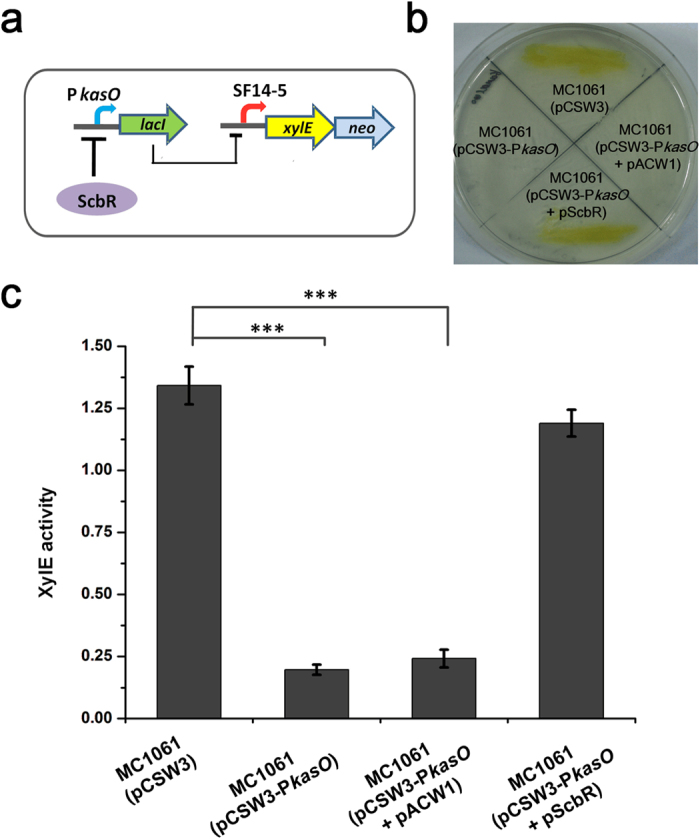
Performance test of the genetic circuit. (**a**) Logic of the genetic circuit in the biosensor. (**b**) Growth and XylE activity of MC1061 harbouring different plasmids on the plate containing 90 μg/ml kanamycin. (**c**) XylE activity of the genetic biosensor in response to different input. The values are presented as mean ± SD of three independent experiments. XylE activity of the strain MC1061 harbouring pCSW3 is set as control. Bridging lines show statistical details of comparisons between the control and the others. Asterisks indicate the statistically significant differences (*p < 0.05; **p < 0.01; ***p < 0.001).

To generate the NOT gate genetic circuit, we constructed the plasmid pCSW3 by inserting the *lacI* gene into the plasmid pCSW2-SF14-5 (Supplementary Fig. S1c). Simultaneously, we introduced a MCS in the upstream of *lacI* to facilitate the insertion of target promoters (Supplementary Fig. S1d). To avoid the interference of native *lacI* from the host genome, we chose the *lacI* deletion mutants of *E. coli* (MC1061 and DH10B) as hosts for the genetic circuit plasmid. To validate the proper function of the genetic circuit, we constructed sensor-plasmid pCSW3-P*kasO* by inserting *kasO* promoter (P*kasO*)^31^ as a target promoter. The MC1061 and DH10B transformants of pCSW3 or pCSW3-P*kasO* were evaluated for their reporter activity, respectively. As expected, the transformants of pCSW3 were able to grow on 90 μg/ml kanamycin with visible XylE activity (Supplementary Fig. S3a), whereas the transformants of pCSW3-P*kasO* did not grow, indicating that LacI expressed from P*kasO* effectively turned off the expression of the reporter cassette. Furthermore, the activity of XylE quantified in MC1061 and DH10B transformants showed that the genetic circuit gave more stringent control in MC1061 (Supplementary Fig. S3b). Therefore, we chose MC1061 as the host of the genetic biosensor. To test the signal conversion of the biosensor, the known repressor of P*kasO*, ScbR^32^, was transformed into MC1061 as a negative input (Fig. 3a). The resulting MC1061(pCSW3-P*kasO* + pScbR) strains restored growth on 90 μg/ml kanamycin and displayed XylE activity (Fig. 3b,c), indicating that the genetic circuit was ideally operated to sense and convert negative input into detectable positive output.

### Evaluation and characterisation of the biosensor

**Figure 4:**
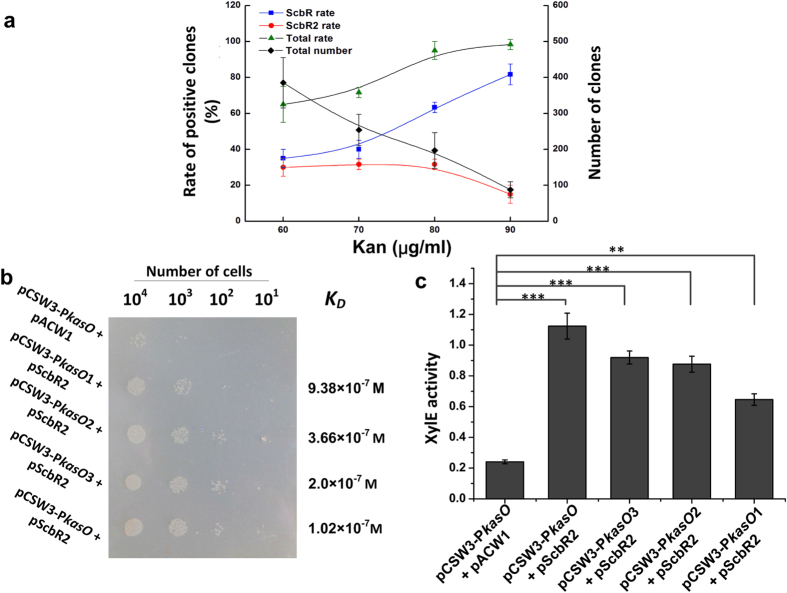
Characterisation of the genetic biosensor. (**a**) Evaluation of the genetic biosensor by an artificial library. The ScbR or ScbR2 rate indicates the positive rate of ScbR or ScbR2 clones identified among the total positive clones, respectively. The total rate is the sum of the ScbR and ScbR2 positive rates. The total numbers indicates the number of visible clones observed on plates. (**b**) Output of kanamycin resistance in response to target promoters with different binding ability. *K*_*D*_ indicates the equilibrium dissociation constant of ScbR2 with the original or mutant *kasO* promoters. The plate contains 90 μg/ml kanamycin. (**c**) Output of XylE activity in response to target promoters with different binding ability. The values are presented as mean ± SD from three independent experiments. XylE activity of the strain with plasmids pCSW3-P*kasO* and pACW1 is set as control. Bridging lines show statistical details of comparisons between the control and the others. Aasterisks indicate the statistically significant differences (*p < 0.05; **p < 0.01; ***p < 0.001)

To evaluate the performance of the genetic biosensor, we used the sensor-plasmid pCSW3-P*kasO* to select the known repressors of P*kasO* (ScbR and ScbR2^31^,^32^) from an artificial repressor library. ScbR and ScbR2 were cloned into the plasmid pACW1. The artificial repressor library (where the ratio of pScbR, pScbR2 and the control pACW1 plasmids was 2:2:96) was introduced into MC1061(pCSW3-P*kasO*) and selected by increasing concentration of kanamycin. As shown in Fig. 4a, the frequency of positive clones (clones containing either pScbR or pScbR2) increased with the kanamycin concentration, suggesting that the biosensor is sensitive. Surprisingly, the frequency of clones harbouring pScbR rose at higher level of kanamycin, whereas that of clones harbouring pScbR2 declined. To ascertain the underlying reason for such differences, XylE activity was tested. The results showed that the repression of P*kasO* by ScbR was stronger than that by ScbR2 (Supplementary Fig. S4). Therefore, the decreasing number of positive clones containing pScbR2 at higher kanamycin levels should be ascribed to the bias of the biosensor, which enriched stronger repressors and reduced the possibility of selecting weaker repressors with increasing kanamycin levels. These results indicate that there is a trade-off between the sensitivity and bias when using the biosensor. Therefore, selection at different kanamycin concentrations can be manipulated to identify diverse repressors of the target promoters.

To evaluate the limit of the binding affinity of the repressors, we decided to characterise the relationship between the equilibrium dissociation constant (*K*_*D*_) of repressors and the output of the genetic biosensor. Since it is hard to find a known promoter having several repressors with different affinity constant, we mutated the operator of P*kasO* (OB site)^23^ to generate four promoters with different binding affinity to the weak repressor ScbR2 (Supplementary Fig. S5a). The *K*_*D*_ of the interaction between these promoters and ScbR2 was measured by electrophoretic mobility shift assay (EMSA)^33^ (Supplementary Fig. S5b–e). Then the output of kanamycin resistance and XylE activity in response to the repression effort of ScbR2 on these target promoters were tested simultaneously. Both results indicated that the genetic biosensor can sense repressors with weak binding ability (Fig. 4b,c); notably, the repression effect of ScbR2 on P*kasO*1 could be significantly reported, although the *K*_*D*_ was nearly 1 μM.

### Selection of the repressors of P*scbA* using the genetic biosensor

To apply the biosensor in the selection of unknown repressors from a repressor library, the promoter P*scbA* of *S. coelicolor* was used as the target (Supplementary Fig. S6a). A repressor library, which contained 63 TetR family regulators of *S. coelicolor*, was constructed using the vector pACW1. To improve the positive rate and avoid the bias of the genetic biosensor, a strict ‘selection–enrichment–selection’ procedure was designed and employed (see Methods). After selection, the positive rate of randomly picked clones was evaluated: XylE activity and the kanamycin resistance level of the clones were compared with the control MC1061(pCSW3-P*scbA* + pACW1), simultaneously. Both tests showed approximate 99% positive rate. To ensure of the saturation of the selection process, the relationship between the number of picked clones and the number of identified repressors was determined (Fig. S7a), where no additional repressors were found when the number of picked clones is more than ten. Hence, four repressors were identified finally: ScbR, ScbR2, Sco6312 and Sco6071. ScbR and ScbR2 are known repressors of P*scbA*^22^,^23^ and Sco6312 and Sco6071 were reported as CprA and CprB previously^34^. However, the current work is the first report of the regulatory relationships between the two regulators CprA, CprB and P*scbA*. The XylE activity of the transformants containing CprA or CprB was also measured, which undoubtedly demonstrated that the two protein turned on the expression of XylE reporter (Supplementary Fig. S8).

### CprA and CprB repress the transcription of P*scbA* by direct binding

**Figure 5:**
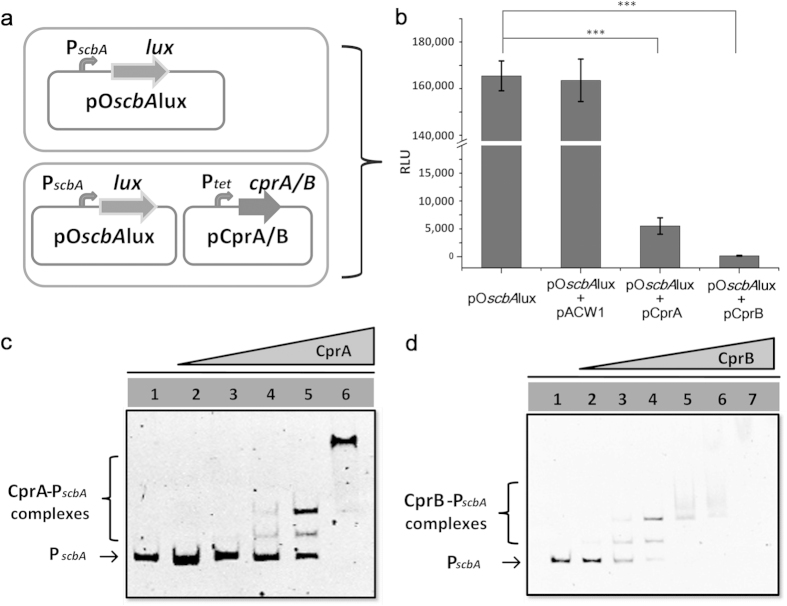
Identification of the selected repressors of P*scbA*. (**a**) Schematic representation of the Lux reporter system. (**b**) CprA and CprB repress the bioluminescence controlled by P*scbA in vivo.* All values are in relative light units (RLU) and represent as mean ± SD from at least three independent experiments. Bioluminescence of the strain with plasmid pO*scbA*lux is set as control. Bridging lines show statistical details of comparisons between the control and the others. Asterisks indicate the statistically significant differences (*p < 0.05; **p < 0.01; ***p < 0.001). (**c**) EMSA of the interaction of P*scbA* with CprA. Each lane contains 6.6 ng of P*scbA* probes and different amounts of CprA. Lanes 1–6 contain 0 nM, 3 nM, 10 nM, 30 nM, 0.1 μM, 0.3 μM of CprA, respectively. (**d**) EMSA of the interaction of P*scbA* with CprB. Each lane contains 6.6 ng of P*scbA* probes and different amounts of CprB. Lanes 1–7 contain 0 nM, 3 nM, 10 nM, 30 nM, 0.1 μM, 0.3 μM and 1 μM of CprB, respectively.

To confirm that the repression of CprA and CprB is caused by direct interaction with P*scbA*, an *in vivo* Lux reporter system was designed in *E. coli* (Fig. 5a). As shown in Fig. 5b, *lux* genes were directly controlled by P*scbA*, endowing the strains with the ability of bioluminescence. When the expression plasmid of CprA or CprB was transformed into the host harbouring P*scbA* driven Lux reporter, the bioluminescence was severely repressed. The result indicated that CprA and CprB indeed repress the expression of P*scbA*. To further confirm the direct interaction of CprA and CprB with P*scbA*, CprA and CprB were expressed and purified from *E. coli* (Supplementary Fig. S9a) and EMSAs were performed with P*scbA*. As shown in Fig. 5c,d, CprA and CprB were found to bind P*scbA* in a concentration-dependent manner. The binding is specific because no retardation was observed with the negative control *hrdB* promoter (P*hrdB*) (Supplementary Fig. S10a). These results demonstrate that CprA and CprB repress P*scbA* by direct interaction.

### Selection and identification of the repressors of P*aco* from a genomic DNA library

**Figure 6:**
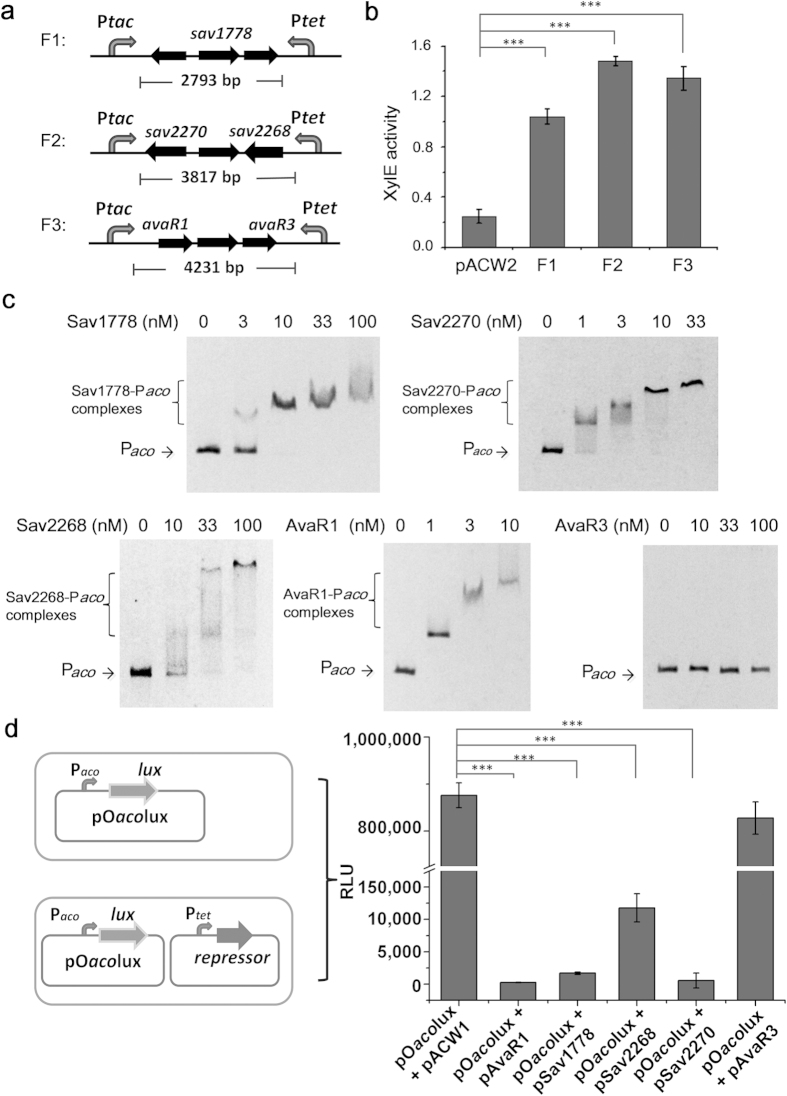
Selection of the repressors of P*aco* from a genomic DNA library. (**a**) Genomic DNA fragments identified by sequencing. (**b**) XylE activity of the biosensor in response to repressors in the three cloned genomic DNA fragments. XylE activity of the strain with plasmid pACW2 is set as control. (**c**) EMSA of the interactions of P*aco* with Sav1778, Sav2270, Sav2268, AvaR1 and AvaR3. Each lane contains 6 ng of P*aco* probes and different amounts of proteins. (**d**) Identification of the repressive roles of Sav1778, Sav2268 and Sav2270 using Lux reporter system in *E. coli*. The values of relative light units (RLU) represent the average of at least three independent experiments. The output of the strain with plasmid pO*aco*lux is set as control. For both (**b**) and (**d**), values are presented as mean ± SD from three independent experiments. Bridging lines show statistical details of comparisons between the control and the others. Asterisks indicate the statistically significant differences (*p < 0.05; **p < 0.01; ***p < 0.001).

To apply the genetic biosensor in the identification of repressors from a genomic library, P*aco* of *S. avermitilis* was used as the target promoter (Supplementary Fig. S6b), and a genomic library containing 3–6 kb fragments of *S. avermitilis* DNA was constructed using the vector pACW2. Approximately 1.7 × 10^5^ clones (based on the number of clones growing on the control plates with only the antibiotics for holding the plasmids) were selected using the improved ‘selection–enrichment–selection’ procedure. The relationship between the number of the picked clones and the number of the identified different fragments is shown in Fig. S7b, indicating the selection is saturated under our experimental conditions. Three different fragments were identified (Fig. 6a). The XylE activity of the three clones was further measured, which confirmed that the products of the three genomic DNA fragments repressed the transcription of P*aco*, and thus inducing the expression of the XylE reporter (Fig. 6b).

The three genomic DNA fragments contain coding sequences of five predicted regulatory proteins: Sav1778, Sav2268, Sav2270, AvaR1 and AvaR3 (Fig. 6a), among which AvaR1 was previously reported as a repressor of P*aco*^24^. To confirm their direct binding to P*aco*, the five identified repressors were expressed and purified from *E. coli* (Supplementary Fig. S9b), and EMSA experiments were performed with the P*aco* probe. As shown in Fig. 6c, the binding of Sav1778, Sav2268, Sav2270 and AvaR1 with P*aco* was detected in a concentration-dependent manner, whereas AvaR3 did not bind P*aco* even when it was provided at high concentrations. The negative control P*hrdB* showed no band-shift when the concentration of these identified repressors reached 1 μM, indicating that these binding interactions were specific. (Supplementary Fig. S10b). Although AvaR1 has been previously reported to bind P*aco*^24^, the repressors Sav1778, Sav2268 and Sav2270 were found to repress P*aco* by direct binding for the first time.

To further confirm the repressive effects of the identified repressors Sav1778, Sav2268 and Sav2270, an *in vivo* test in heterologous host *E. coli* was performed, which can display clear results without the influence of any indirect regulations in native host (Fig. 6d). The empty plasmid (pACW1) and AvaR3 were tested as the negative controls and displayed no influence on the activity of P*aco*; while the positive control AvaR1 and newly identified Sav1778, Sav2268 and Sav2270 showed significant repression effect on the expression of Lux reporter driven by P*aco*. These data combining our *in vitro* EMSA results (Fig. 6c) strongly suggest that the newly identified proteins are true repressors.

## Discussion

Gene expression is orchestrated by complex transcriptional regulatory networks to fine-tune physiological responses. To define a regulatory network, it is necessary to determine specific TFs capable of binding to target promoters. However, efficient methods for genome-wide detection of repressors that directly bind target promoters in bacteria are still underdeveloped, which severely limits the efforts to dissect transcriptional regulatory networks. Herein, a NOT gate genetic circuit was designed, built and applied in *E. coli* as a genetic biosensor for the selection and identification of repressors. The systemic characterisation and practical applications of the genetic biosensor indicate that this method is applicable for identifying genome-wide unknown repressors of promoters.

There are two significant advantages of the genetic biosensor method. One is that it is readily accessible: only a simple selection procedure is required to obtain clones containing repressors, and only basic molecular biology expertise is required to identify the repressors of promoters of interest. The other is that this method can efficiently identify the direct-binding repressors of promoters in *E. coli*, thereby avoiding the false positive of the disturbance of indirect repressors by intrinsic regulatory networks in the native host. Compared to other *in vitro* methods for identifying regulators of target promoters, such as DNA affinity capture, which relies on the relative abundance of TFs in a given sample and expensive facilities^10^,^11^,^12^,^13^,^14^, this genetic biosensor based strategy provides an efficient method to specifically identify the repressors of target promoters. Since the repressors selected by the biosensor are expressed in a heterologous host, this strategy can potentially avoid the problem of differential expression of regulators in DNA affinity capture experiments.

The genetic biosensor method requires the efficient expression of the candidate repressors. Otherwise the false negative rate is expected to increase. Similar problems have been experienced in the modified bacterial one-hybrid system, which requires the combined expression of the DNA binding domain of candidate TFs with the α subunit of RNA polymerase in *E. coli*^9^. To efficiently construct libraries for selection by the genetic biosensor, the specialised plasmids pACW1 and pACW2 were designed for the repressor library and genomic library, respectively (Fig. 1b,c); and the In-Fusion cloning strategy (Clontech) or AT cloning strategy was employed. By combining all these techniques and strategies, the genetic biosensor method can promise efficient selection of repressors of target promoters.

Despite the importance of signalling molecules in the regulation of cellular functions in streptomycetes, the control of signalling molecule production is not well understood at systemic level^21^. To dissect the underlying mechanisms of the temporal control of these signalling molecules, the first step is to determine the genome-wide repressors acting on the promoter regions of signalling molecule synthase genes. Therefore, the usefulness of the genetic biosensor method was proven by the identification of multiple repressors of P*scbA* and P*aco* in *S. coelicolor* and *S. avermitilis*, respectively. All the newly identified repressors of P*scbA* and P*aco* are TetR family regulators, suggesting that TetR family repressors play important roles in controlling signalling molecule production in *S. coelicolor* and *S. avermitilis*.

In prokaryotes, complex regulation occurs at the promoter regions of key genes, such as the promoter regions of cluster-situated regulators in antibiotic synthesis clusters and the promoter region of key developmental regulatory gene *adpA* in *Streptomyces*^20^,^35^. This study offers an innovative and powerful genetic biosensor to dissect unknown repressors in such complex regulation. In addition, this strategy can serve as a reference to identify repressors in eukaryotes. Similar to the development of two-hybrid system: enlightened by the yeast two-hybrid system^36^, a number of bacterial two-hybrid systems have been developed^37^,^38^,^39^. In conclusion, we developed a genetic biosensor from a *de novo* designed genetic circuit, which provides a convenient tool to identify genome-wide unknown repressors of promoters of interest. This work will facilitate the dissection of the transcriptional regulatory networks in prokaryotes.

## Methods

### Bacterial strains and growth conditions

The strains and plasmids used in this study are listed in Supplementary Table S1. *E. coli* JM109 and DH5α were used for cloning and luciferase assay, respectively; *E. coli* BL21(DE3) was used for the recombinant expression of the identified repressors; *E. coli* MC1061 and MC4100 were used as the hosts for testing the genetic circuit. *S. coelicolor* M145 and *S. avermitilis* NRRL 8165 were cultivated in supplemented minimum medium (SMM) and yeast extract-malt extract medium, respectively^26^. The *E. coli* strains were grown in Luria-Bertani (LB) containing ampicillin (100 μg/ml), hygromycin (50 μg/ml), kanamycin (25 μg/ml) or chloramphenicol (25 μg/ml) when necessary.

### Construction of plasmids

A set of plasmids for genetic circuit design and evaluation were constructed as follows. All the primers used are listed in Supplementary Table S2. To construct pDR4-P*tac*, the P*tac* fragment obtained by overlap PCR using primers Ptac-F/Ptac-R was digested by BglII and SpeI, and then inserted into similarly digested pDR4^26^. To replace the replication origin of pDR4-P*tac*, the replication origin of pSC101 was amplified from pCS26-Pac^31^ using primers pSC-F/pSC-R, and a pDR4-P*tac* fragment without the pUC replication origin was amplified using primers PDR-F/PDR-R. The two fragments were ligated by In-Fusion cloning kit (Clontech) to construct pCSW1. Afterwards, pCSW1 was digested by XbaI and NheI to remove the 3402 bp fragment containing the integration elements of *Streptomyces*, and the remaining 4807 bp was blunt-ended and self-ligated to construct pCSW2. To construct pCSW2-SF14, pCSW2-SF14-1, pCSW2-SF14-2, pCSW2-SF14-3, pCSW2-SF14-4 and pCSW2-SF14-5, the SF14 promoter and its variants were obtained by overlap PCR using primers SF14F/ SF14R, SF14F/ SF14-1R, SF14-2F/ SF14R, SF14-2F/SF14-1R, SF14-4F/ SF14-4R and SF14F/ SF14-5R, respectively. These promoter fragments were digested by BglII and SpeI and ligated with similarly digested pCSW2. To construct pCSW3, the *lacI* gene from pET28a (Novagen) and a transcription terminator (*tfd*) from pIJ8660^40^ were amplified using primers lacIqF/lacIqR and tfdF/tfdR, respectively; these fragments were ligated to the plasmid pCSW2-SF14-5 digested by BamHI and BsrGI by In-Fusion cloning kit (Clontech). To construct pCSW3-P*kasO*, *kasO* promoter was amplified by primers kasOpF/kasOpR from *S. coelicolor* genome; then the PCR products were digested by appropriate enzymes and inserted in BamHI and ScaI digested pCSW3. To obtain the plasmids for library construction, a fragment from pScbR^26^ was obtained by PCR using primers 184F/184R and self-ligated to generate pACW1. Another fragment amplified with primers ACWF/ACWR from pScbR^26^ was self-ligated to generate pACW2. The complete sequences of pCSW3, pACW1 and pACW2 are presented in the Supplementary Information.

### Evaluation of kanamycin-resistance levels and XylE activity

To evaluate the kanamycin-resistance levels, the *E. coli* strains were spread out on plates containing different concentrations of kanamycin (Kan) and then incubated at 37 °C for 12 h. The kanamycin resistance level was determined as the concentration on which poor growth was observed, whereas the higher concentration completely inhibited growth. To obtain a more accurate assessment of the kanamycin resistance level, the strains were diluted to equal number of cells, and spotted onto LB plates with different concentrations of kanamycin.

To observe the catechol-2, 3-dioxygenase (XylE) activity on plates, colonies were grown on agar plates for 12 h, then sprayed with an aqueous solution of catechol (0.5 M) and incubated for 5 min at 37 ^o^C. Quantitative measurement of total catechol-2, 3-dioxygenase (XylE) activity in cell-free extracts was performed as described previously^41^. XylE activity was measured during the exponential growth phase (approximately 12 h). The XylE activity was calculated as the rate of change in optical density at 375 nm per minute per OD_600_ of the culture. All assays were performed in triplicate. Data are presented as mean ± standard derivation (SD). Statistical significance were calculated using a one-way analysis of variance (ANOVA) followed by Tukey post-hoc test, and *p-*values < 0.05 were considered statistically significant. All statistical analyses were performed with R statistical software (v3.2.0).

### Evaluation of the synthetic biosensor for selection of repressors

To evaluate the behaviour of the biosensor, the sensor-plasmid pCSW3-P*kasO* was introduced into MC1061. An artificial regulator library is composed of the plasmid pACW1, and two repressor-expressing plasmids pScbR and pScbR2, which were constructed by inserting *scbR* and *scbR2* into pACW1^23^,^31^; within the library, the ratio of pACW1, pScbR and pScbR2 was adjusted to 96:2:2. Then the library was introduced into MC1061 containing pCSW3-P*kasO* and the transformants were selected by kanamycin resistance levels. The clones on plates containing 60 μg/ml, 70 μg/ml, 80 μg/ml or 90 μg/ml of kanamycin were counted. The total number is the number of visible clones grown under the selection condition. The frequencies of the appearance of pScbR or pScbR2 were obtained by sequencing 25 clones randomly picked from each plate. The clone numbers of pScbR or pScbR2 divided by 25 gave the positive rates of clones containing pScbR or pScbR2, respectively. The total positive rate is the sum of positive rates of pScbR and pScbR2.

To obtain a series of promoters with different affinity to ScbR2, the OB site of P*kasO* was mutated using primers KOPF1/KOPR, KOPF2/KOPR, KOPF3/KOPR. The equilibrium dissociation constant (*K*_*D*_) was characterised by electrophoretic mobility shift assay as previous reported^33^. The relative abundance of bound probes was measured by gray-scale assays. To construct the sensor plasmids containing the mutant promoters, the plasmid fragments were amplified with the primers KOPF1/KOER1, KOPF2/KOER2 and KOPF3/KOER3 using pCSW3-P*kasO* as template and self-ligated via Gibson assembly method^42^ to generate pCSW3-P*kasO*1, pCSW3-P*kasO*2 and pCSW3-P*kasO*3, respectively. These plasmids and pScbR2 were transformed into MC1061, then the XylE activity and kanamycin resistance were measured.

### Selection for the repressors of P*scbA* and P*aco*

To construct pCSW3-P*scbA*, P*scbA* was amplified using primers scbApF/scbApR from *S. coelicolor* M145 genomic DNA; the PCR product was digested by BamHI and inserted into pCSW3 digested with BamHI and ScaI. Similarly, P*aco* was amplified using primers acoF/acoR from *S. avermitilis* genomic DNA and inserted into pCSW3 to generate pCSW3-P*aco*. pCSW3-P*scbA* and pCSW3-P*aco* were transformed into MC1061. To construct a TetR repressor library of *S. coelicolor* M145, the fragment of pACW1 was obtained by PCR using primers 184F/184R. A total of 63 genes of annotated TetR family regulators in *S. coelicolor* genome were amplified by high-fidelity PCR using primers in Supplementary Table S2; each fragment contained 15 bp sequences at two ends complementary to the fragment of pACW1. Then the 63 TetR repressor fragments were mixed to give equal concentration of each fragment, and ligated with the fragment of pACW1 by In-Fusion cloning technology (Clontech). To construct the genomic DNA library of *S. avermitilis*, the genomic DNA was fractured to isolate 3–6 kb fragments, then an adenine residue was added to the 3′ ends of these fragments using Taq DNA polymerase. The pACW2 plasmid was treated with XcmI to generate a linear fragment with one thymine at 3′ ends. The genomic fragments were cloned into the linearized pACW2 by TA cloning.

To detect the repressors, an improved strict “selection–enrichment–selection” procedure was developed. First, the transformants were selected on LB agar with 75 μg/ml kanamycin. Then the positive clones were pooled and enriched in liquid LB with 75 μg/ml kanamycin for 6 h. Finally, the strains from the enrichment culture were selected again by spreading on LB agar with 85 μg/ml kanamycin. The positive clones were identified by sequencing.

### Expression and purification of the repressors

The genes *cprA* and *cprB* were amplified from the genomic DNA of *S. coelicolor* M145 with primers cprAF/cprAR and cprBF/cprBR; and *avaR1*, *avaR3*, *sav1778*, *sav2268* and *sav2270* were amplified from the genomic DNA of *S. avermitilis* using primers R1F/R1R, R3F/R3R, 78F/78R, 68F/68R and 70F/70R, respectively. The amplified fragments were cloned into pET23b treated by NdeI and XhoI via Gibson assembly method^42^ to obtain the plasmids pET-CprA, pET-CprB, pET-AvaR1, pET-AvaR3, pET-Sav1778, pET-Sav2268 and pET-Sav2270. The plasmids were introduced into *E. coli* BL21(DE3) for recombinant expression. The purification of these proteins, as well as the expression and purification of ScbR2, was performed as previously described^23^.

### Electrophoretic mobility shift assay (EMSA)

The probes P*scbA* and P*aco* were amplified with the primers ScbApF/ScbApR and acoF/acoR, respectively. The subsequent binding experiments were performed using a modified gel mobility shift assay previously described^23^.

### Construction of Lux reporter systems and luciferase assays *in vivo*

The plasmid pO*scbA*lux containing P*scbA*-controlled *lux* reporter genes was constructed previously^31^. To construct pO*aco*lux, P*aco* was amplified with primers EacoF/EacoR, then trimmed by XhoI and BamHI and inserted into pCS26-Pac. For the expression of identified repressors in the Lux reporter system, pCprA and pCprB were isolated and identified from the TetR repressor library; *sav1778*, *sav2268*, *sav2270*, *avaR1* and *avaR3* were amplified from the genomic DNA of *S. avermitilis* using primers E78F/E78R, E68F/E68R, E70F/E70R, ER1F/ER1R and ER3F/ER3R, respectively. The amplified fragments were cloned into pACW1 by In-Fusion cloning technology (Clontech) to generate pSav1778, pSav2268, pSav2270 pAvaR1 and pAvaR3, respectively. For luciferase assays, pCprA and pCprB were introduced into DH5α harbouring pO*scbA*lux; pSav1778, pSav2268, pSav2270 pAvaR1 and pAvaR3 were introduced into DH5α harbouring pO*aco*lux. After 12 h of incubation, bioluminescence of the *E. coli* cultures was measured with a single tube luminometer (Turner Biosystems). All assays were performed in triplicate. Data are presented as mean ± SD. Statistical significance were calculated using a one-way ANOVA followed by Tukey post-hoc test, and *p-*values < 0.05 were considered statistically significant.

## Additional Information

**How to cite this article**: Wang, W. *et al.* A genetic biosensor for identification of transcriptional repressors of target promoters. *Sci. Rep.* **5**, 15887; doi: 10.1038/srep15887 (2015).

## Supplementary Material

Supplementary Information
