# Supplementary material for: A genetic biosensor for identification of transcriptional repressors of target promoters
Source: Sci Rep. 2015 Oct 29;5:15887. doi: 10.1038/srep15887 (PMC4625125; doi:10.1038/srep15887)
Supplement: Supplementary Information [file srep15887-s1.pdf]

Supplementary information

A genetic biosensor for identification of transcriptional repressors of target promoters

Weishan Wang[+], Xiao Li[+], Yue Li, Shanshan Li, Keqiang Fan and Keqian Yang*

State Key Laboratory of Microbial Resources, Institute of Microbiology, Chinese Academy of Sciences, Beijing, 100101, People's Republic of China

* To whom correspondence should be addressed. E-mail: yangkq@im.ac.cn.

[+] These authors contributed equally to this work.

Supplementary Figures:

Figure S1 Key sequences or maps of plasmids pACW1, pACW2 and pCSW3.

Figure S2 Performance of P*tac*-controlled *xylE-neo* reporter cassette.

Figure S3 Performance of genetic circuits in *E. coli* MC1061 and DH10B.

Figure S4 Output of XylE activity in response to known repressors ScbR or ScbR2.

Figure S5 Evaluation of the binding affinity of the *kasO* derived promoters and ScbR2.

Figure S6 Sequences of P*scbA* and P*aco* promoters used for identifying repressors.

Figure S7 Evaluation of the saturated selection of P*scbA* and P*aco*.

Figure S8 XylE activity of the biosensor in response to CprA or CprB.

Figure S9 Purification of identified repressors.

Figure S10 EMSA of the interactions between identified repressors and the negative control promoter.

Supplementary Tables:

Table S1 Strains and plasmids used in this study.

Table S2 Primers and synthetic oligonucleotides used in this study.

Supplementary sequences of pACW1, pACW2 and pCSW3

Reference

Supplementary Figures:

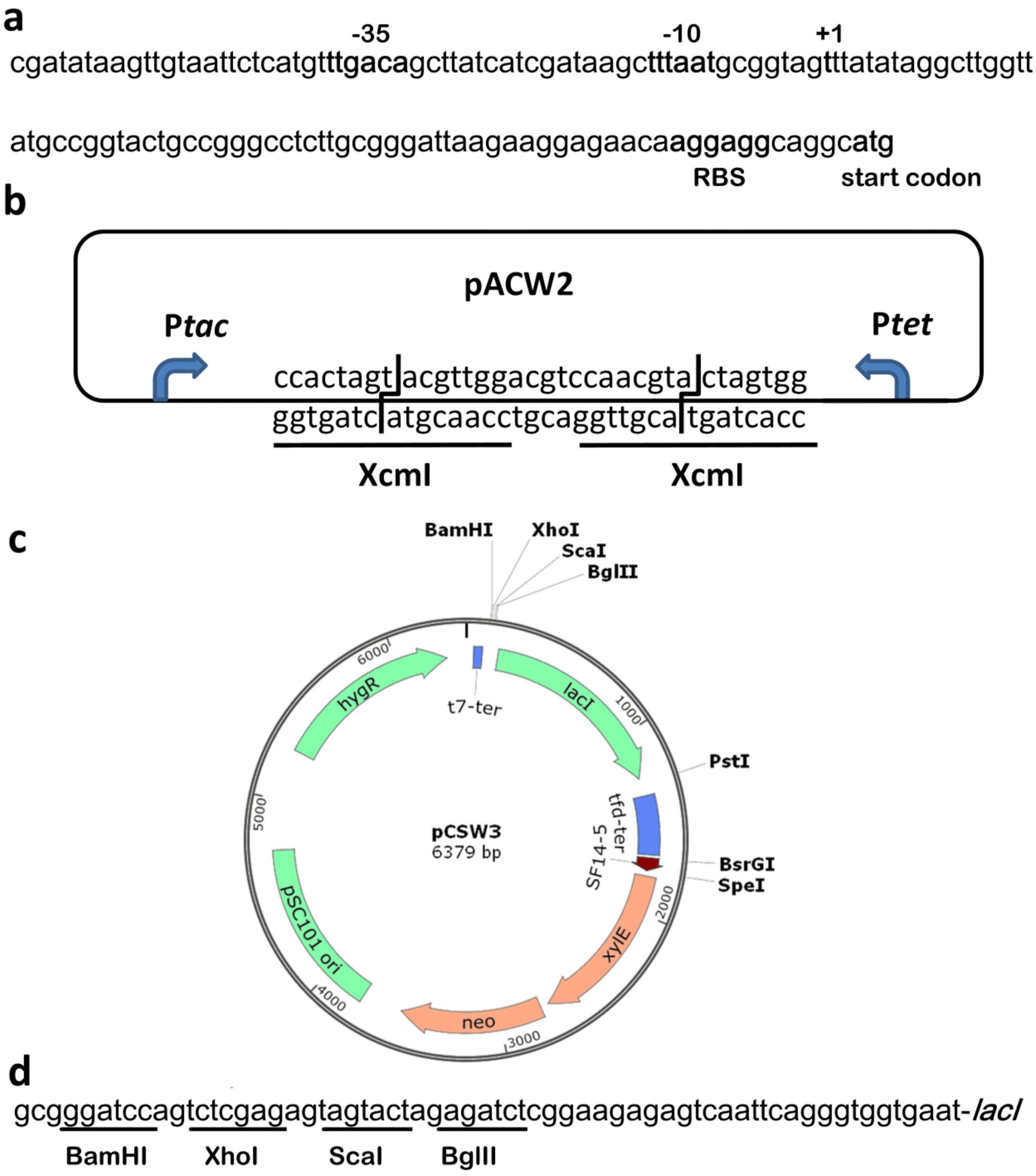

Supplementary Figure S1 Key sequences or maps of plasmids pACW1, pACW2 and pCSW3. (**a**) Sequence of P*tet* promoter and RBS of pACW1 for constructing repressor library. (**b**) Schematic of genomic library plasmid pACW2, highlighting the positions of P*tac* and P*tet* promoters and the two XcmI restriction sites for AT cloning. (**c**) Map of genetic circuit plasmid pCSW3. *hygR*, hygromycin resistance gene; tfd-ter, major transcription terminator of phage fd; T7-ter, transcription terminator of phage T7; pSC101 ori, origin of replication of pSC101. (**d**) Multiple clone sites of pCSW3 used for inserting target promoters.

a

ggcaaatattctgaaatgagctg**ttgaca**attaatcatcggctcg**tataat**gtgtgg aattgtgagcggataacaatttcacac

-35 -10 +1 *lacO*

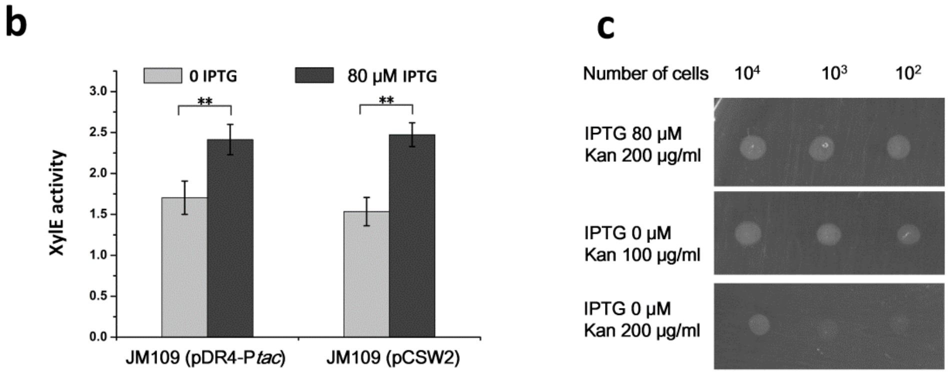

Supplementary Figure S2 Performance of P*tac*-controlled *xylE-neo* reporter cassette. (**a**) Sequence of P*tac.* The -35 and -10 sites of the promoter are shown in bold letters and the *lacO* operator is underlined. The sign +1 indicates the transcription start site. (**b**) Activity of XylE under the control of P*tac* in *E. coli* JM109 with or without IPTG. The plasmid pCSW2 has a low copy number replication origin from pSC101. The values are presented as mean ± SD from three independent experiments. XylE activity of the strains without IPTG introduction is set as control. Bridging lines show statistical details of comparisons between the controls and the others. Asterisks indicate the statistically significant differences (* $p < 0.05$; ** $p < 0.01$; *** $p < 0.001$). (**c**) Activity of P*tac* in *E. coli* JM109(pCSW2) reported by kanamycin resistance levels on LB plate.

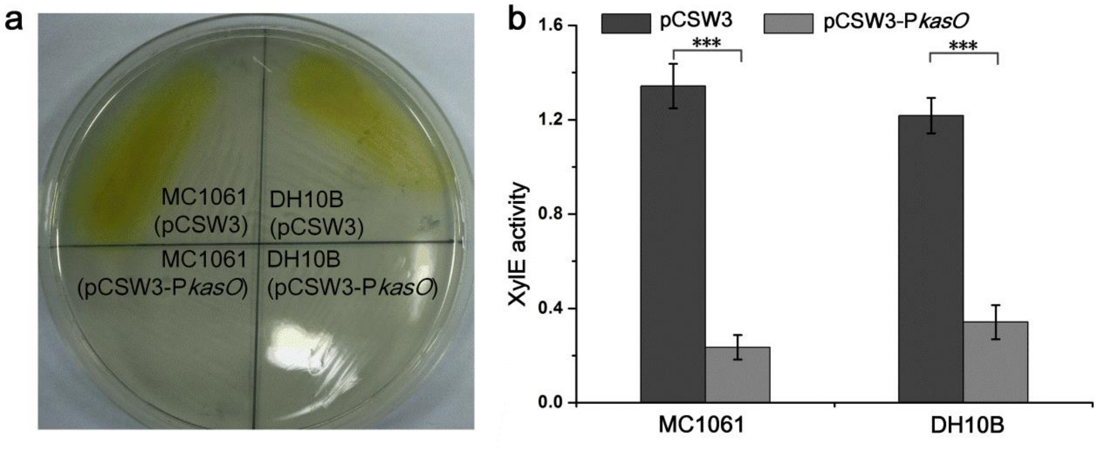

Supplementary Figure S3 Performance of genetic circuits in *E. coli* MC1061 and DH10B. (**a**) Growth and XylE activity of strains on plate with 90 μg/ml kanamycin. (**b**) XylE activity of strains in liquid LB medium. The columns and bars indicate mean ± SD. XylE activity of strains harboring pCSW3 is set as control. Bridging lines show statistical details of comparisons between the controls and the others. Asterisks indicate the statistically significant differences (* $p < 0.05$; ** $p < 0.01$; *** $p < 0.001$).

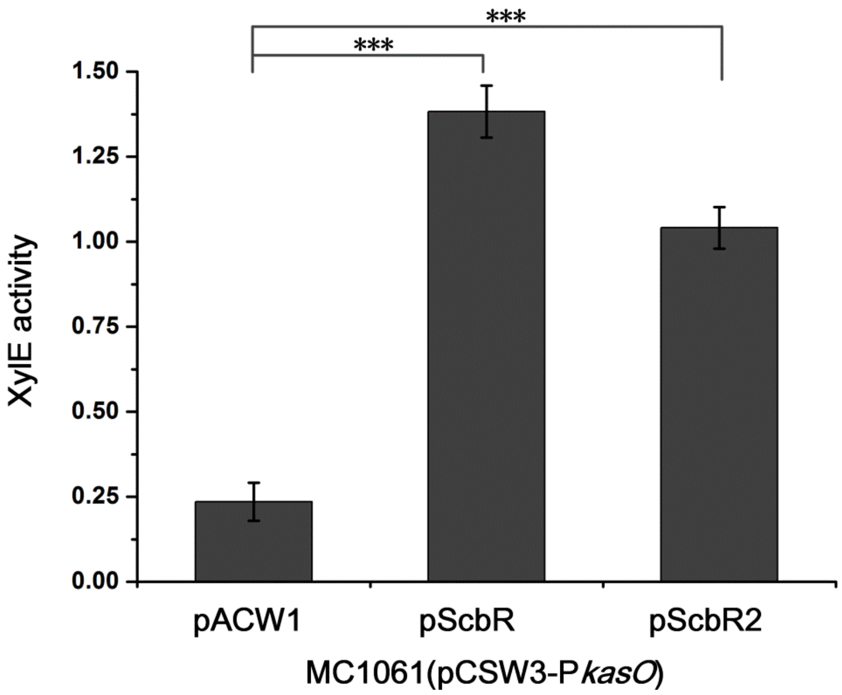

Supplementary Figure S4 Output of XylE activity in response to known repressors ScbR or ScbR2**.** The values are presented as mean ± SD. XylE activity of MC1061(pCSW3-P*kasO* + pACW1) is set as control. Bridging lines show statistical details of comparisons between the control and the others. Asterisks indicate the statistically significant differences (* $p < 0.05$; ** $p < 0.01$; *** $p < 0.001$).

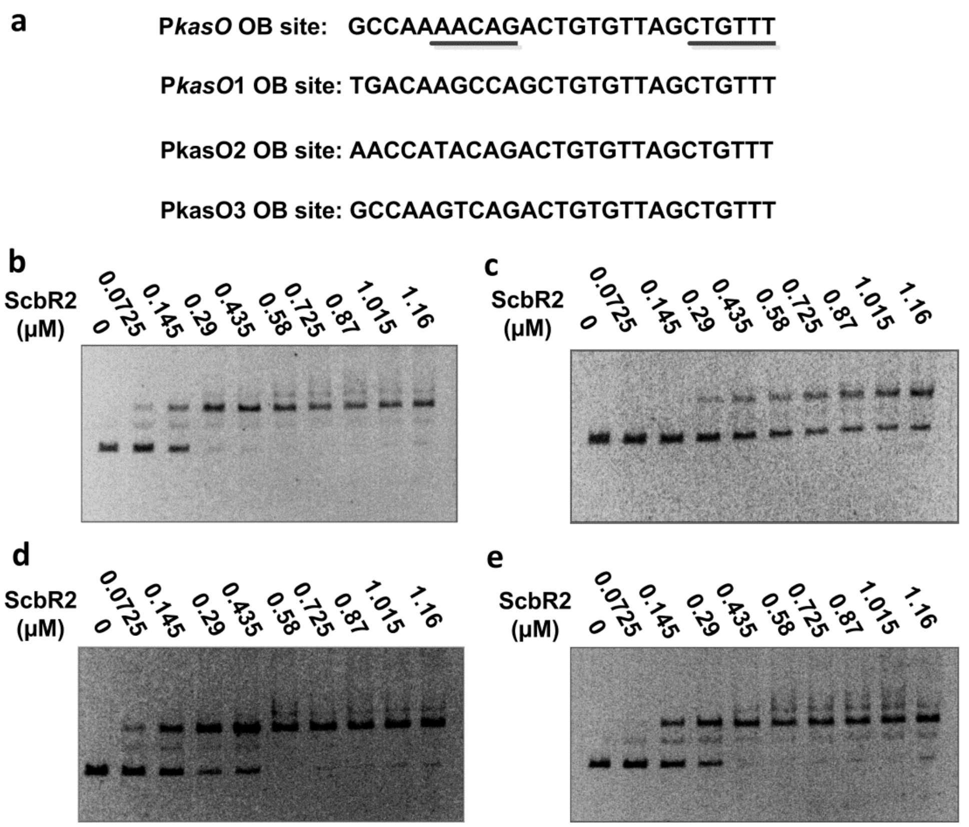

Supplementary Figure S5 Evaluation of the binding affinity of the *kasO* derived promoters and ScbR2. (**a**) Sequences of original and mutant OB sites of *kasO* promoters (P*kasO*). The palindromic sequence of OB site is underlined. (**b**), (**c**), (**d**) and (**e**) EMSA results of ScbR2 with P*kasO*, P*kasO*1, P*kasO*2 and P*kasO*3, respectively. Each lane contains 6.2 ng of P*kasO* probes and different amounts of proteins

a

tgaggatctccgtgatcgtggcagcttggtagccctgcttctcgaagacctgcgccgc*gg
cgtccaggatcgtctgccgcgtgcggatcgcccggtcctgcttggccatgcctgcctcctt
gttcatgtctcccccgggaaggа**tagaaa**aaaaaccgctcagtctg**tatctt**aacgttcg
cgcatacagaacagctcggcatcacatgattcctgggggggacccatg

b

acgcaccctccgcaccagcccgaccagcgggacaccgtcccgaccagtgggacac
cgtccggatcgcgggacaccgtcccgaccagcgggaaaagaccaagcggttcgca
ccctaacagcgacgatcctccactccaccgccgaagacaggcccggtcctgaccgg
gacgataaaaccagctgaacggtttttcaatgctcccctcgccctctcacaaaccactca
aaaacacatatctcacccccaactcccttgtaagggatgacccttgaccgcaagaggg
acttgaagacaaaaccgtctagtacgtatctttgacctccagctcttccgcagctcttccgc
agctcctccgcagctcttccgcagctctccagcccttcctcagcccttctcgggtcccgtg
gttcacgtcagcccgtcccaagccagggagaagctcgatg

Supplementary Figure S6 Sequences of P*scbA* and P*aco* promoters used for identifying repressors. (**a**) Sequence of P*scbA*. The -10 and-35 regions are underlined and the transcription start site is marked with an asterisk. (**b**) Sequence of P*aco*. As -35 and -10 sequences of P*aco* are unknown, this fragment is chosen because it is upstream of the *aco* coding sequence.

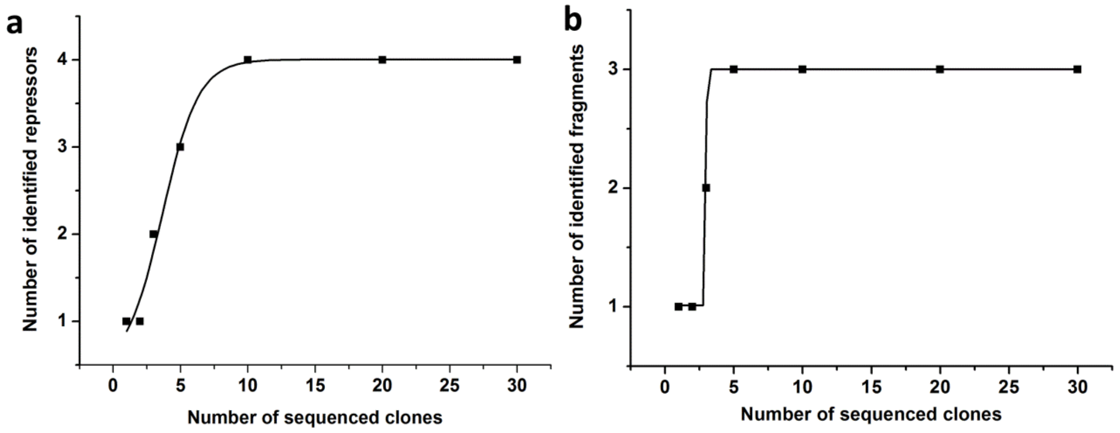

Supplementary Figure S7 Evaluation of the saturation of the selection. (**a**) Relationship between the number of picked clones and the number of identified repressors for selecting repressors of P*scbA*. (**b**) Relationship between the number of picked clones and the number of identified different fragments for selecting repressors of P*aco*.

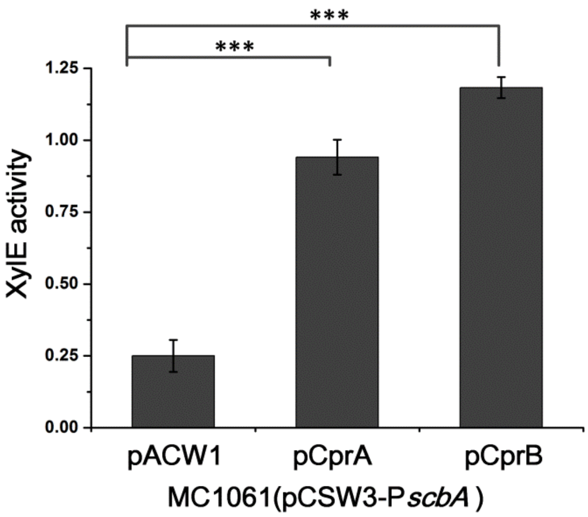

Supplementary Figure S8 XylE activity of the biosensor in response to CprA or CprB. The values are presented as mean ± SD from three independent experiments. XylE activity of MC1061(pCSW3-P*scbA* + pACW1) is set as control. Bridging lines show statistical details of comparisons between the control and the others. Asterisks indicate the statistically significant differences (* $p < 0.05$; ** $p < 0.01$; *** $p < 0.001$).

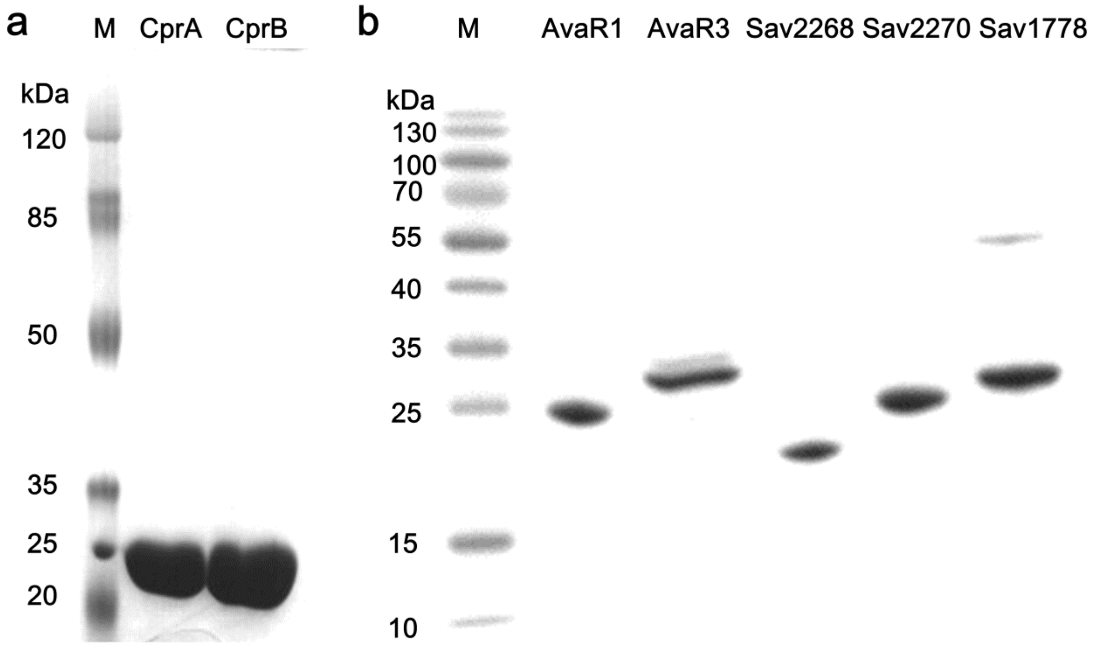

Supplementary Figure S9 Purification of identified repressors. (**a**) SDS-PAGE result showing the purified CprA and CprB. (**b**) SDS-PAGE result showing the purified AvaR1, AvaR3, Sav2268, Sav2270 and Sav1778. Lane M is the protein marker.

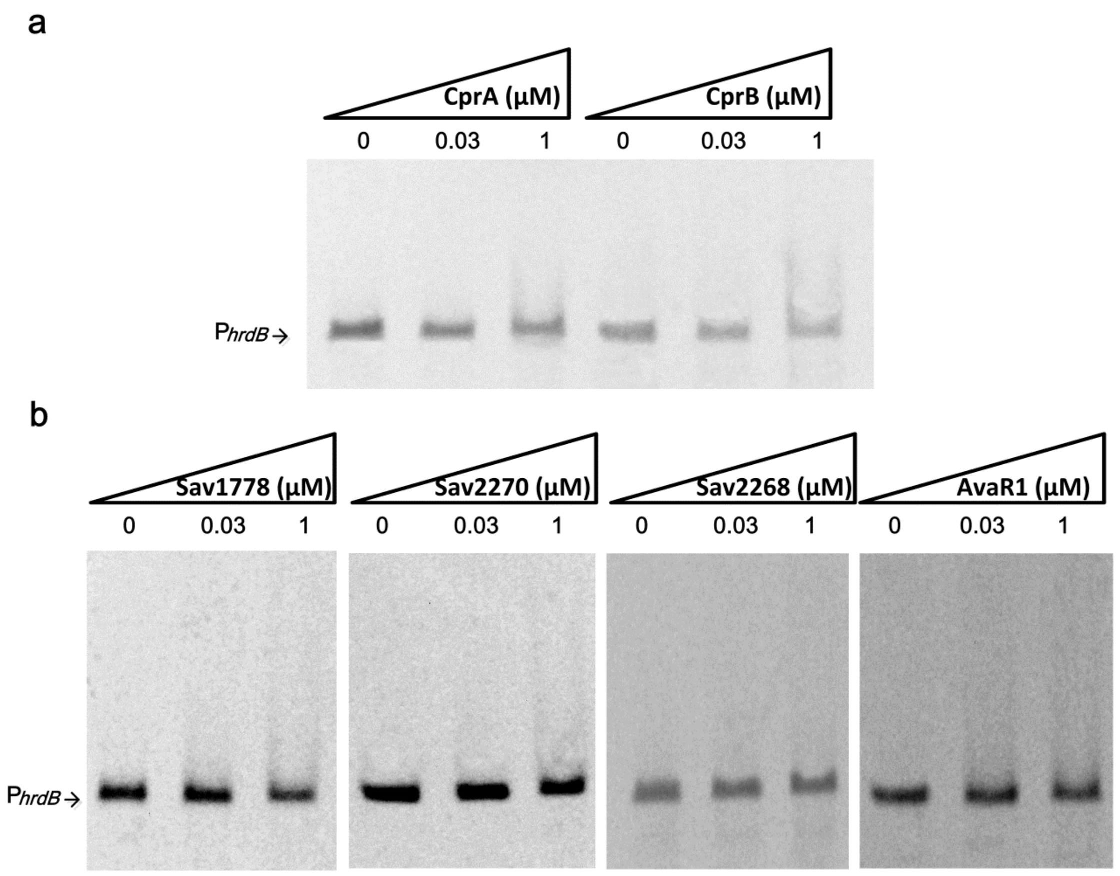

Supplementary Figure S10 EMSA of the interactions between identified repressors and the negative control promoter. (**a**) EMSA of the interactions between CprA or CprB and the negative control promoter. (**b**) EMSA of the interactions between Sav1778, Sav2270, Sav2268 or AvaR1 and the negative control promoter. Here, the promoter region of *hrdB* (P*hrdB*) was chosen as negative control. Each lane contains 6 ng of P*hrdB* probes and different amounts of identified repressor proteins.

Supplementary Tables:

Supplementary Table S1 Strains and plasmids used in this study.

Name	Relevant genotypes	Sources
Streptomyces		
S. coelicolor M145	A prototrophic derivative of *S. coelicolor* A3(2)	John Innes[1]
S. avermitilis NRRL 8165	Wild type	ATCC
E.coli		
JM109	General cloning host for plasmid manipulation	Novagen
DH5α	Host for reporter system	Novagen
BL21 (DE3)	Host for expression plasmids with T7 derived promoter	Novagen
MC1061	Host for genetic circuits	Novagen
DH10B	Host for genetic circuits	Novagen
plasmids		
pDR4	Apramycin resistance gene replaced by hygromycin resistance gene in pDR3	Our lab[2]
pDR4-P*tac*	Insert P*tac* into pDR4	This study
pCS26-Pac	Kan^R, promoterless *luxCDABE* reporter, pSC101*ori*	Our lab[3]
pCSW1	The replication origin of pDR4-P*tac* is replaced by the one of pSC101	This study

pCSW2	Remove the integration elements of *Streptomyces* from pCSW1	This study
pCSW2-SF14	Insert SF14 into pCSW2	This study
pCSW2-SF14-1	Insert SF14-1 into pCSW2	This study
pCSW2-SF14-2	Insert SF14-2 into pCSW2	This study
pCSW2-SF14-3	Insert SF14-3 into pCSW2	This study
pCSW2-SF14-4	Insert SF14-4 into pCSW2	This study
pCSW2-SF14-5	Insert SF14-5 into pCSW2	This study
pIJ8660	Contain the major transcription terminator of phage *tfd*	Sun et.al.[4]
pCSW3	Insert *lacI* and *tfd* terminator into pCSW2-SF14-5	This study
pCSW3-P*kasO*	Insert *kasO* promoter into the upstream of *lacI* gene in pCSW3	This study
pCSW3-P*kasO*1	Mutate P*kasO* to P*kasO*1 in pCSW3-P*kasO*	This study
pCSW3-P*kasO2*	Mutate P*kasO* to P*kasO*2 in pCSW3-P*kasO*	This study
pCSW3-P*kasO3*	Mutate P*kasO* to P*kasO*3 in pCSW3-P*kasO*	This study
pCSW3-P*scbA*	Insert *scbA* promoter into the upstream of *lacI* gene in pCSW3	This study
pCSW3-P*aco*	Insert *aco* promoter into the upstream of *lacI* gene in pCSW3	This study
pACYC184	Cm^R , Tc^R, contain the replication origin of p15A	NEB
pACW1	For repressor library construction, contain	This study

	appropriate RBS downstream of P*tet*	
pACW2	For genomic library construction, Insert P*tac* and two XcmI sites into pACYC184	This study
pO*scbA*lux	pCS26-Pac containing the *scbA* promoter region	Our lab[5]
pO*aco*lux	pCS26-Pac containing the *aco* promoter region	This study
pSav1778	For the expression of Sav1778 in Lux reporter system	This study
pSav2268	For the expression of Sav2268 in Lux reporter system	This study
pSav2270	For the expression of Sav2270 in Lux reporter system	This study
pAvaR1	For the expression of AvaR1 in Lux reporter system	This study
pAvaR3	For the expression of AvaR3 in Lux reporter system	This study
pScbR	Cm^R , for the expression of ScbR	Our lab[2]
pScbR2	Cm^R , for the expression of ScbR2	Our lab[5]
pET23b	Expression vector with T7 promoter, contain N-terminal T7 tag and C-terminal His_6-tag	Novagen
pET-CprA	For the expression CprA, *cprA* is inserted into pET23b	This study
pET-CprB	For the expression CprB, *cprB* is inserted into pET23b	This study
pET-AvaR1	For the expression AvaR1, *avaR1* is inserted into	This study

	pET23b	
pET-AvaR3	For the expression AvaR3, *avaR3* is inserted into pET23b	This study
pET-Sav1778	For the expression Sav1778, *sav1778* is inserted into pET23b	This study
pET-Sav2268	For the expression Sav2268, *sav2268* is inserted into pET23b	This study
pET-Sav2270	For the expression Sav2270, *sav2270* is inserted into pET23b	This study

Supplementary Table S2 Primers and synthetic oligonucleotides used in this study.

Primers	Sequence (5′-3′)[a]	Restriction enzymes
Ptac-F	CAGTAGATCTGTACAGGCAAATATTCTGAAATGAGCTGTTGACAATTAATC	BglII, BsrGI
Ptac-R	CAGTACTAGTCCACACATTATACGAGCCGATGATTAATTGTCAACAG	SpeI
PCS-F	GCCAGCTGCATTAATTTGTCTCAGGTGTTCAATTTCATG	No
PCS-R	CTCTTTTTCCGAAGGTCGGAGACAAAAGGAATATTC	BamHI
PXN-F	TCCTTTTGTCTCCGACCTTCGGAAAAAGAGTTGGTAG	No
PXN-R	GAACACCTGAGACAAATTAATGCAGCTGGCACGAC	No
SF14F	CAGTAGATCTGTACAGCCTTGACCTTGATGAGGCGGCGTGAGCTACAAT	BglII, BsrGI
SF14R	CAGTACTAGTCTAATCGAGTATTGATTGTAGCTCACGCCGCCTC	SpeI
SF14-1R	CAGTACTAGTGAATTGTTATCCGCTCACAATTCTAATCGAGTATTGATTGTAGCTCACGCCGCCTC	SpeI
SF14-2F	CAGTAGATCTGTACAAATTGTGAGCGGATAACAA	BglII,

	TTGCCTTGACCTTGATGAGGCGGCGTGAGCTAC	BsrGI
SF14-4F	CAGTAGATCTGTACAGCCTTGACCTTAACAATAGGCGAGTGTTAACAATC	BglII, BsrGI
SF14-4R	CAGTACTAGTCTTAACAATAGGCGAGTGTTAACAATCAATACTCGATTAG	SpeI
SF14-5R	CAGTACTAGTGAATTGTTATCCGCTCACAATTCGAGTATTGATTGTAGCTCACGCCGCCTCATC	SpeI
lacIqF	TATATCCGCGGATCCAGTCTCGAGAGTAGTACTAGAGATCTCGGAAGAGAGTCAATTCAGGGTG	BamHI, XhoI,ScaI, BglII
lacIqR	GCTGCAGCGCACATTAATTGCGTTGCGCTCACTGCC	No
tfdF	AATGTGCGCTGCAGCCCGTACCTCTAGTAGAGTCGAG	No
tfdR	GGTCAAGGCATGTACATCTAGCTAGAGGATCTAAAGTTTTGTC	No
kasOpF	GCGGGATCCGACGAGGAGATCGACCG	BamHI
kasOpR	ACTCTCGAGCGAGTGGACCGAGCATC	XhoI
KOPF1	GGTGACAAGCCAGCTGTGTTAGCTGTTT	No
KOPR	TATTCTCCTGGCCACGACTTTAC	No
KOPF2	GGGCCAAGTCAGACTGTGTTAGCTGTTT	No
KOPF3	GGAACCATACAGACTGTGTTAGCTGTTT	No

KOER1	AACAGCTAACACAGCTGGCTTGTCACCGTCCGGC GACCGCCGGGACGGTC	No
KOER2	AACAGCTAACACAGTCTGACTTGGCCCGTCCGGC GACCGCCGGGACGGTC	No
KOER3	AACAGCTAACACAGTCTGTATGGTTCCGTCCGGCG ACCGCCGGGACGGTC	No
ScbApF	GCG<u>GGATCC</u>TGAGGATCTCCGTGATCGTG	BamHI
ScbApR	TCAGCTACTTATGCACCCTGGTCCGGTGGAC	No
acoF	GCG<u>GGATCC</u>GCACCCTCCGCACCAGCC	BamHI
acoR	CGAGCTTCTCCCTGGCTTGG	No
184-F	CGACATCACCGATGGGGAAGATC	No
184-R	ATGCCTGCCTCCTTGTTCTCCTTC	No
ACWF	CATCGGCTCGTATAATGTGTGGCCACTAGTACGTT GGACGTCCAACGTACTAGTGGACACGGTGCCTGA CTGCGTTAG	No
ACWR	CCACACATTATACGAGCCGATGATTAATTGTCAAC AGCTCATTTCAGAATATTTGCCCTCGCTAACGGAT TCACCACTC	No
SCO6286F	CAAGGAGGCAGGCATATGACCAAGCAGGAGCGG GCGGCAC	No
SCO6286R	CCATCGGTGATGTCGTCAGTGCGGCGCGTCCTGCC GCTC	No

SCO6265F	CAAGGAGGCAGGCATATGGCCAAGCAGGACCGGG CGATC	No
SCO6266R	CCATCGGTGATGTCGTCAGTCCTTCCCGGTCGGTG C	No
SCO6312F	CAAGGAGGCAGGCATATGGCGAGGCAGCTACGCG CGGAAC	No
SCO6312R	CCATCGGTGATGTCGTCAGGTCGTGCCCGTCTCCT GTTC	No
SCO6071F	CAAGGAGGCAGGCATATGGCGAGGCAGCTACGCG CCGAG	No
SCO6071R	CCATCGGTGATGTCGTCAGGCCGTGCCCGTCTCCT G	No
SCO2100F	CAAGGAGGCAGGCATATGGAAAGCAGCAGCACCG CC	No
SCO2100R	CCATCGGTGATGTCGACGGACCGGCGTTCTTCGTG C	No
SCO3367F	CAAGGAGGCAGGCATATGAACGGCACCAAGCAGC AG	No
SCO3367R	CCATCGGTGATGTCGGCAAGGGCGTGACCGTCTG AC	No
SCO5170F	CAAGGAGGCAGGCATATGACAGCCATCGAGCAGA CAGA	No

SCO5170R	CCATCGGTGATGTCGGCCGTACCAGCACACATTAGC	No
SCO6071F	CAAGGAGGCAGGCATATGGCGAGGCAGCTACGCGCCGAG	No
SCO6071R	CCATCGGTGATGTCGGACGATCAGCCCGGGCACGAC	No
SCO6312F	CAAGGAGGCAGGCATATGGCGAGGCAGCTACGCGCGGAACG	No
SCO6312R	CCATCGGTGATGTCGCACCGCCGTCCTGCTGACCGGCTTC	No
SCO6323F	CAAGGAGGCAGGCATATGCAGGAACGGGCGAAGGC	No
SCO6323R	CCATCGGTGATGTCGGAGCCGTTACCACAACGAAG	No
SCO1691F	CAAGGAGGCAGGCATATGACCCCCGCCGGGGCGCAC	No
SCO1691R	CCATCGGTGATGTCGGTACGCGGGCGCCCGGACGCTGATG	No
SCO4499F	CAAGGAGGCAGGCATATGGGTGCAGGGAAGACCAAGC	No
SCO4499R	CCATCGGTGATGTCGCACGGCCGTACGAAGCCAAGTAC	No

SCO4385F	CAAGGAGGCAGGCATATGAACGCGGCCGACCGCAC	No
SCO4385R	CCATCGGTGATGTCGCACCGACAGTGCCCCGGTGC	No
SCO3833F	CAAGGAGGCAGGCATATGACCACCGCCAAGCGCGA	No
SCO3833R	CCATCGGTGATGTCGTCAGGACGCCTTGCGCAACC	No
SCO6781F	CAAGGAGGCAGGCATATGACTGAACCGCATGGTCC	No
SCO6781R	CCATCGGTGATGTCGCTCAACAAGGGCTCCTCCGG	No
SCO4850F	CAAGGAGGCAGGCATATGCCTGCGACCAACGACGG	No
SCO4850R	CCATCGGTGATGTCGGGCGACACCCAGCTCTACGTC	No
SCO4480F	CAAGGAGGCAGGCATATGAGAGCGTGCCTGCCCAT	No
SCO4480R	CCATCGGTGATGTCGGATCGTCACCCCGCGGTGGA	No
SCO6474F	CAAGGAGGCAGGCATATGTCCCAGCCCGCCAAGTC	No
SCO6474R	CCATCGGTGATGTCGGAATCATCTCCCGCCGAAGG	No
SCO1699F	CAAGGAGGCAGGCATATGAGTACGGCGGCCGAGAC	No
SCO1699R	CCATCGGTGATGTCGAGGTGAGCGTCG	No

SCO1702F	CAAGGAGGCAGGCATATGGCCCGACCGCGCAAGC	No
SCO1702R	CCATCGGTGATGTCGCATGCGTCCAGGATGCGCCAC	No
SCO0485F	CAAGGAGGCAGGCATATGCCCGACTCACCGACCCCAG	No
SCO0485R	CCATCGGTGATGTCGTGCTGCGCGATCCGGAGGCG	No
SCO4952F	CAAGGAGGCAGGCATATGAAGGACGGCGAAGCGGC	No
SCO4952R	CCATCGGTGATGTCGGAGTTGCCCCAGTTCCGTCAG	No
SCO0646F	CAAGGAGGCAGGCATATGAGCTCACCGGAACCCCC	No
SCO0646R	CCATCGGTGATGTCGGTCGCACCCGCACGGTCCAC	No
SCO0310F	CAAGGAGGCAGGCATATGGACATGAAGCCCGAAGACC	No
SCO0310R	CCATCGGTGATGTCGCTCCGCTCGTTCGGAATCAG	No
SCO4303F	CAAGGAGGCAGGCATATGGAGACGCGACAGAAGGG	No
SCO4303R	CCATCGGTGATGTCGGGAGGAGGTGCTGAAGGCGC	No
SCO7809F	CAAGGAGGCAGGCATATGAGTGAGCAGCAACCAA	No

	AC	
SCO7809R	CCATCGGTGATGTCGCAGCGATTGTTCAGCACTTC	No
SCO2374F	CAAGGAGGCAGGCATATGAAGGTGGCCGGCACGTC	No
SCO2374R	CCATCGGTGATGTCGGTACGCGCCAGACGTTCAGC	No
SCO6784F	CAAGGAGGCAGGCATATGAGCACCGAGCAGAGCCA	No
SCO6784R	CCATCGGTGATGTCGGAAGCGGGTACGGGCCTGGA	No
SCO3979F	CAAGGAGGCAGGCATATGACCGTGCAGCAGTCGGG	No
SCO3979R	CCATCGGTGATGTCGCTTAGGCCGCTCGGGCAACC	No
SCO7694F	CCAAGGAGGCAGGCATATGAGCAGGAGCGACGCCGG	No
SCO7694R	CCATCGGTGATGTCGCTACCACGTCACCCTCCACG	No
SCO5532F	CAAGGAGGCAGGCATATGGGTCCGCAGGAGAAGCC	No
SCO5532R	CCATCGGTGATGTCGGACGTCCCGCGACCGTCGAT	No
SCO0728F	CAAGGAGGCAGGCATATGAGCACCAGCAGTGCGGC	No
SCO0728R	CCATCGGTGATGTCGGTGCACGCACCACAGCGTAT	No
SCO1904F	CAAGGAGGCAGGCATATGCCCGGAAGGTTGCCCG	No

	C	
SCO1904R	CCATCGGTGATGTCGGTCCGCGGCATGGTGAAGCC	No
SCO5209F	CAAGGAGGCAGGCATATGCCCGCAGCCCGGGAGTC	No
SCO5209R	CCATCGGTGATGTCGACTAGGGATCCTCCCCTGCG	No
SCO6702F	CAAGGAGGCAGGCATATGACATCGACCGCCGCCAG	No
SCO6702R	CCATCGGTGATGTCGCTCACCCGCAGCGGCCGAAG	No
SCO3346F	CAAGGAGGCAGGCATATGAACGGCGACGCCGGCGG	No
SCO3346R	CCATCGGTGATGTCGGTCTCGTCGTACGCCGTCGGCG	No
SCO4977F	CAAGGAGGCAGGCATATGACCGCCGCACGTTCCGC	No
SCO4977R	CCATCGGTGATGTCGCGAACAGGTGCAGCTCGGGG	No
SCO1034F	CAAGGAGGCAGGCATATGGGGAGTACCGCGCCGGC	No
SCO1034R	CCATCGGTGATGTCGGTGGCCGGTGCGGGCCACCC	No

SCO3201F	CAAGGAGGCAGGCATATGGACATGAAGCCCGAAGACC	No
SCO3201R	CCATCGGTGATGTCGCAGCTCATCGCGAGATCGAT	No
SCO0857F	CAAGGAGGCAGGCATATGACTGGCCAGGCGGCCGG	No
SCO0857R	CCATCGGTGATGTCGGAGGAAGCCCGCAGGGGCGA	No
SCO3769F	CAAGGAGGCAGGCATATGACCGTCGGCGCACGCGA	No
SCO3769R	CCATCGGTGATGTCGGTCATCCCGGGGGAGACGGTCGG	No
SCO4167F	CAAGGAGGCAGGCATATGCCGCCGGGTGGTCGCAG	No
SCO4167R	CCATCGGTGATGTCGCGACGAGTGGTGGGACGAACTGG	No
SCO0337F	CAAGGAGGCAGGCATATGGCAAGGTGGGACCCGGG	No
SCO0337R	CCATCGGTGATGTCGGCTGCACGAGGAGTCGGTCA	No
SCO0520F	CAAGGAGGCAGGCATATGTCCGACGCCACGAAACG	No
SCO0520R	CCATCGGTGATGTCGCTCCACGTACAGCTCCAGCG	No

SCO0521F	CAAGGAGGCAGGCATATGGGTCGATGGGAGCCGAACGC	No
SCO0521R	CCATCGGTGATGTCGCTGTTCCGCCTCGTCGGCGG	No
SCO0302F	CAAGGAGGCAGGCATATGACGCAACACCGTGTTCC	No
SCO0302R	CCATCGGTGATGTCGCCTCTACAGCTTCGCCGCGC	No
SCO4661F	CAAGGAGGCAGGCATATGACCGGCAGCACCCGCGG	No
SCO4661R	CCATCGGTGATGTCGGAAGTCGACCCCGGCAACGCC	No
SCO6792F	CAAGGAGGCAGGCATATGACCACGGCCGCGAAGACCG	No
SCO6792R	CCATCGGTGATGTCGGAGCGTGCGGTGCGGCTGGC	No
SCO4871F	CAAGGAGGCAGGCATATGAGTACGGAACGACGCGCC	No
SCO4871R	CCATCGGTGATGTCGGCTCGTCACGCGTGCGGTAC	No
SCO5483F	CAAGGAGGCAGGCATATGAGTCACACCCCCGGTGT	No
SCO5483R	CCATCGGTGATGTCGGTCTGGGACAGCACCGACCG	No
SCO6784F	CAAGGAGGCAGGCATATGAGCACCGAGCAGAGCC	No

	A	
SCO6784R	CCATCGGTGATGTCGGTGTCCAGTACGGCCCACACC	No
SCO0155F	CAAGGAGGCAGGCATATGACGAGCAGTACCGGGACG	No
SCO0155R	CCATCGGTGATGTCGCAACTGGTGCCCCACGGTCA	No
SCO6778F	CAAGGAGGCAGGCATATGTCCGAGCAGCTGAACGC	No
SCO6778R	CCATCGGTGATGTCGGTAATGGTGTCTGCGGTCATTGC	No
SCO1193F	CAAGGAGGCAGGCATATGGCACAGCGGCGCCGCGG	No
SCO1193R	CCATCGGTGATGTCGCTCATCCGCTCCCGCGTCGTGG	No
SCO7539F	CAAGGAGGCAGGCATATGCCCACAGAGCGACCCAC	No
SCO7539R	CCATCGGTGATGTCGCGAGATCGAGGCACTGTCCGC	No
SCO6121F	CAAGGAGGCAGGCATATGTCGGTACAGGAACGCAA	No
SCO6121R	CCATCGGTGATGTCGGACTAGGACCTCGCTGAGCA	No

SCO7518F	CAAGGAGGCAGGCATATGGCAACCGGACACACGGA	No
SCO7518R	CCATCGGTGATGTCGGACGGCGTCTGCTGACAGGG	No
SCO2243F	CAAGGAGGCAGGCATATGTCGGTACAGGAACGCAAGC	No
SCO2243R	CCATCGGTGATGTCGCTGTGGAAGGTCCCGACGAA	No
SCO4030F	CAAGGAGGCAGGCATATGACAGTAGCGGAGGACCC	No
SCO4030R	CCATCGGTGATGTCGGTCCACGCGAACATCGCCGG	No
SCO0669F	CAAGGAGGCAGGCATATGACGCCCAACCGTCACAACC	No
SCO0669R	CCATCGGTGATGTCGGACGGTGTCGGCCACCTCGC	No
SCO0508F	CAAGGAGGCAGGCATATGGCGCTCGCCGGTGAGGT	No
SCO0508R	CCATCGGTGATGTCGGTCGACTGCCCGAGCACCCC	No
cprAF	TTGTTTAACTTTAAGAAGGAGATATACATATGGCGAGGCAGCTACGCGC	No
cprAR	TCTCAGTGGTGGTGGTGGTGGTGCTCGAGGGTCGTGCCCGTCTCCTG	No

cprBF	TTGTTTAACTTTAAGAAGGAGATATACATATGGCGAGGCAGCTACGCGCCGAG	No
cprBR	TCTCAGTGGTGGTGGTGGTGGTGCTCGAGGGCCGTGCCCGTCTCCTG	No
R1F	TCTCAGTGGTGGTGGTGGTGGTGCTCGAGCTCCAACGTCCGTGCCGCGTCC	No
R1R	TTGTTTAACTTTAAGAAGGAGATATACATATGGCGCGGCAGGAGCGAGCCATTC	No
R2F	AGCCGGATCTCAGTGGTGGTGGTGGTGGTGCTCGAGTCCGGCGGTGGTCGCCGGGGTC	No
R2R	TGTTTAACTTTAAGAAGGAGATATACATATGACGAAACAGGAACGCGCCG	No
R3F	ATCTCAGTGGTGGTGGTGGTGGTGCTCGAGGCGCGTCTCCGGCGGCCTGCCCGAC	No
R3R	ATTTTGTTTAACTTTAAGAAGGAGATATACATATGGTCAAGCAGGTCAGACAGG	No
78F	TCTCAGTGGTGGTGGTGGTGGTGCTCGAGGTCCGCGGAGCGGTCCGCCGTC	No
78R	ATTTTGTTTAACTTTAAGAAGGAGATATACATATGACCAGCGTCGACGAACCG	No
68F	ATCTCAGTGGTGGTGGTGGTGGTGCTCGAGGCGGGCCGGGGCGCCGCCGAGCGCCTG	No

68R	TAATTTTGTTTAACTTTAAGAAGGAGATATACATATGGAGCCGGCAAAGGAGGGGAG	No
70F	TCTCAGTGGTGGTGGTGGTGGTGCTCGAGGTGCAGTGGCACGAAGGCGTC	No
70R	TAATTTTGTTTAACTTTAAGAAGGAGATATACATATGGACGTCATGAGTAGCGAG	No
EacoF	TCAC<u>CTCGAG</u>GCACCCTCCGCACCAGCCCGAC	XhoI
EacoR	TAGA<u>GGATCC</u>CGAGCTTCTCCCTGGCTTGGGAC	BamHI
E78F	CAAGGAGGCAGGCATATGACCAGCGTCGACGAACCG	No
E78R	CCATCGGTGATGTCGTCAGTCCGCGGAGCGGTCCGCCGTC	No
E68F	CAAGGAGGCAGGCATGTGGAGCCGGCAAAGGAGGGGAG	No
E68R	CCATCGGTGATGTCGTCAGCGGGCCGGGGCGCCGCCGAG	No
E70F	CAAGGAGGCAGGCATATGGACGTCATGAGTAGCGAGC	No
E70R	CCATCGGTGATGTCGTCAGTGCAGTGGCACGAAGGC	No
ER1F	CAAGGAGGCAGGCATGTGGCGCGGCAGGAGCGAGC	No

ER1R	CCATCGGTGATGTCGTCACTCCAACGTCCGTGCCGC	No
ER3F	CAAGGAGGCAGGCATATGGTCAAGCAGGTCAGACAG	No
ER3R	CCATCGGTGATGTCGTCAGCGCGTCTCCGGCGGCCTG	No

[a]The restriction enzyme sites of primers are underlined.

Supplementary sequences of pACW1, pACW2 and pCSW3:

pACW1 (4012 bp):

gaattccggatgagcattcatcaggcgggcaagaatgtgaataaaggccggataaaacttgtgcttatttttctttacggtcttt
aaaaaggccgtaatatccagctgaacggtctggttataggtacattgagcaactgactgaaatgcctcaaaatgttctttacg
atgccattgggatatatcaacggtggtatatccagtgatttttttctccattttagcttccttagctcctgaaaatctcgataactca
aaaaatacgcccggtagtgatcttatttcattatggtgaaagttggaacctcttacgtgccgatcaacgtctcattttcgccaaa
agttggcccagggcttcccggtatcaacagggacaccaggatttatttattctgcgaagtgatcttccgtcacaggtatttattc
ggcgcaaagtgcgtcgggtgatgctgccaacttactgatttagtgtatgatggtgtttttgaggtgctccagtggcttctgtttct
atcagctgtccctcctgttcagctactgacggggtggtgcgtaacggcaaaagcaccgccggacatcagcgctagcggag
tgtatactggcttactatgttggcactgatgagggtgtcagtgaagtgcttcatgtggcaggagaaaaaaggctgcaccggt
gcgtcagcagaatatgtgatacaggatatattccgcttcctcgctcactgactcgctacgctcggtcgttcgactgcggcgag
cggaaatggcttacgaacggggcggagatttcctggaagatgccaggaagatacttaacagggaagtgagagggccgc
ggcaaagccgtttttccataggctccgcccccctgacaagcatcacgaaatctgacgctcaaatcagtggtggcgaaaccc
gacaggactataaagataccaggcgtttccccctggcggctccctcgtgcgctctcctgttcctgcctttcggtttaccggtgt
cattccgctgttatggccgcgtttgtctcattccacgcctgacactcagttccgggtaggcagttcgctccaagctggactgta
tgcacgaaccccccgttcagtccgaccgctgcgccttatccggtaactatcgtcttgagtccaacccggaaagacatgcaa
aagcaccactggcagcagccactggtaattgatttagaggagttagtcttgaagtcatgcgccggttaaggctaaactgaaa
ggacaagttttggtgactgcgctcctccaagccagttacctcggttcaaagagttggtagctcagagaaccttcgaaaaacc
gccctgcaaggcggttttttcgttttcagagcaagagattacgcgcagaccaaaacgatctcaagaagatcatcttattaatca
gataaaatatttctagatttcagtgcaatttatctcttcaaatgtagcacctgaagtcagccccatacgatataagttgtaattctc
atgtttgacagcttatcatcgataagctttaatgcggtagtttatcacagttaaattgctaacgcagtcaggcaccgtgtatgaa
atctaacaatgcgctcatcgtcatcctcggcaccgtcaccctggatgctgtaggcataggcttggttatgccggtactgccgg
gcctcttgcgggattaagaaggagaacaaggaggcaggcatcgacatcaccgatggggaagatcgggctcgccacttcg
ggctcatgagcgcttgtttcggcgtgggtatggtggcaggccccgtggccgggggactgttgggcgccatctccttgcatg
caccattccttgcggcggcggtgctcaacggcctcaacctactactgggctgcttcctaatgcaggagtcgcataagggag
agcgtcgaccgatgcccttgagagccttcaacccagtcagctccttccggtgggcgcggggcatgactatcgtcgccgca
cttatgactgtcttctttatcatgcaactcgtaggacaggtgccggcagcgctctgggtcattttcggcgaggaccgctttcgc
tggagcgcgacgatgatcggcctgtcgcttgcggtattcggaatcttgcacgccctcgctcaagccttcgtcactggtcccg
ccaccaaacgtttcggcgagaagcaggccattatcgccggcatggcggccgacgcgctgggctacgtcttgctggcgttc
gcgacgcgaggctggatggccttccccattatgattcttctcgcttccggcggcatcgggatgcccgcgttgcaggccatg
ctgtccaggcaggtagatgacgaccatcagggacagcttcaaggatcgctcgcggctcttaccagcctaacttcgatcact
ggaccgctgatcgtcacggcgatttatgccgcctcggcgagcacatggaacgggttggcatggattgtaggcgccgccct
ataccttgtctgcctccccgcgttgcgtcgcggtgcatggagccgggccacctcgacctgaatggaagccggcggcacct
cgctaacggattcaccactccaagaattggagccaatcaattcttgcggagaactgtgaatgcgcaaaccaacccttggca
gaacatatccatcgcgtccgccatctccagcagccgcacgcggcgcatctcgggcagcgttgggtcctggccacgggtg
cgcatgatcgtgctcctgtcgttgaggacccggctaggctggcggggttgccttactggttagcagaatgaatcaccgatac
gcgagcgaacgtgaagcgactgctgctgcaaaacgtctgcgacctgagcaacaacatgaatggtcttcggtttccgtgtttc
gtaaagtctggaaacgcggaagtcccctacgtgctgctgaagttgcccgcaacagagagtggaaccaaccggtgatacc
acgatactatgactgagagtcaacgccatgagcggcctcatttcttattctgagttacaacagtccgcaccgctgtccggtag
ctccttccggtgggcgcggggcatgactatcgtcgccgcacttatgactgtcttctttatcatgcaactcgtaggacaggtgc
cggcagcgcccaacagtcccccggccacggggcctgccaccatacccacgccgaaacaagcgccctgcaccattatgtt
ccggatctgcatcgcaggatgctgctggctaccctgtggaacacctacatctgtattaacgaagcgctaaccgtttttatcag

gctctgggaggcagaataaatgatcatatcgtcaattattacctccacggggagagcctgagcaaactggcctcaggcattt
gagaagcacacggtcacactgcttccggtagtcaataaaccggtaaaccagcaatagacataagcggctatttaacgaccc
tgccctgaaccgacgaccgggtcgaatttgctttcgaatttctgccattcatccgcttattatcacttattcaggcgtagcacca
ggcgtttaagggcaccaataactgccttaaaaaaattacgccccgccctgccactcatcgcagtactgttgtaattcattaag
cattctgccgacatggaagccatcacagacggcatgatgaacctgaatcgccagcggcatcagcaccttgtcgccttgcgt
ataatatttgcccatggtgaaaacgggggcgaagaagttgtccatattggccacgtttaaatcaaaactggtgaaactcaccc
agggattggctgagacgaaaaacatattctcaataaaccctttagggaaataggccaggttttcaccgtaacacgccacatc
ttgcgaatatatgtgtagaaactgccggaaatcgtcgtggtattcactccagagcgatgaaaacgtttcagtttgctcatggaa
aacggtgtaacaagggtgaacactatcccatatcaccagctcaccgtctttcattgccatacg

pACW2 (3128 bp):

gaattccggatgagcattcatcaggcgggcaagaatgtgaataaaggccggataaaacttgtgcttatttttctttacggtcttt
aaaaaggccgtaatatccagctgaacggtctggttataggtacattgagcaactgactgaaatgcctcaaaatgttctttacg
atgccattgggatatatcaacggtggtatatccagtgatttttttctccattttagcttccttagctcctgaaaatctcgataactca
aaaaatacgcccggtagtgatcttatttcattatggtgaaagttggaacctcttacgtgccgatcaacgtctcattttcgccaaa
agttggcccagggcttcccggtatcaacagggacaccaggatttatttattctgcgaagtgatcttccgtcacaggtatttattc
ggcgcaaagtgcgtcgggtgatgctgccaacttactgatttagtgtatgatggtgtttttgaggtgctccagtggcttctgtttct
atcagctgtccctcctgttcagctactgacggggtggtgcgtaacggcaaaagcaccgccggacatcagcgctagcggag
tgtatactggcttactatgttggcactgatgagggtgtcagtgaagtgcttcatgtggcaggagaaaaaaggctgcaccggt
gcgtcagcagaatatgtgatacaggatatattccgcttcctcgctcactgactcgctacgctcggtcgttcgactgcggcgag
cggaaatggcttacgaacggggcggagatttcctggaagatgccaggaagatacttaacagggaagtgagagggccgc
ggcaaagccgtttttccataggctccgcccccctgacaagcatcacgaaatctgacgctcaaatcagtggtggcgaaaccc
gacaggactataaagataccaggcgtttccccctggcggctccctcgtgcgctctcctgttcctgcctttcggtttaccggtgt
cattccgctgttatggccgcgtttgtctcattccacgcctgacactcagttccgggtaggcagttcgctccaagctggactgta
tgcacgaaccccccgttcagtccgaccgctgcgccttatccggtaactatcgtcttgagtccaacccggaaagacatgcaa
aagcaccactggcagcagccactggtaattgatttagaggagttagtcttgaagtcatgcgccggttaaggctaaactgaaa
ggacaagttttggtgactgcgctcctccaagccagttacctcggttcaaagagttggtagctcagagaaccttcgaaaaacc
gccctgcaaggcggttttttcgttttcagagcaagagattacgcgcagaccaaaacgatctcaagaagatcatcttattaatca
gataaaatatttctagatttcagtgcaatttatctcttcaaatgtagcacctgaagtcagccccatacgatataagttgtaattctc
atgtttgacagcttatcatcgataagctttaatgcggtagtttatcacagttaaattgctaacgcagtcaggcaccgtgtccact
agtacgttggacgtccaacgtactagtggccacacattatacgagccgatgattaattgtcaacagctcatttcagaatatttg
ccctcgctaacggattcaccactccaagaattggagccaatcaattcttgcggagaactgtgaatgcgcaaaccaacccttg
gcagaacatatccatcgcgtccgccatctccagcagccgcacgcggcgcatctcgggcagcgttgggtcctggccacgg
gtgcgcatgatcgtgctcctgtcgttgaggacccggctaggctggcggggttgccttactggttagcagaatgaatcaccg
atacgcgagcgaacgtgaagcgactgctgctgcaaaacgtctgcgacctgagcaacaacatgaatggtcttcggtttccgt
gtttcgtaaagtctggaaacgcggaagtcccctacgtgctgctgaagttgcccgcaacagagagtggaaccaaccggtga
taccacgatactatgactgagagtcaacgccatgagcggcctcatttcttattctgagttacaacagtccgcaccgctgtccg
gtagctccttccggtgggcgcggggcatgactatcgtcgccgcacttatgactgtcttctttatcatgcaactcgtaggacag
gtgccggcagcgcccaacagtcccccggccacggggcctgccaccatacccacgccgaaacaagcgccctgcaccatt
atgttccggatctgcatcgcaggatgctgctggctaccctgtggaacacctacatctgtattaacgaagcgctaaccgtttttta
tcaggctctgggaggcagaataaatgatcatatcgtcaattattacctccacggggagagcctgagcaaactggcctcagg
catttgagaagcacacggtcacactgcttccggtagtcaataaaccggtaaaccagcaatagacataagcggctatttaacg
accctgccctgaaccgacgaccgggtcgaatttgctttcgaatttctgccattcatccgcttattatcacttattcaggcgtagc
accaggcgtttaagggcaccaataactgccttaaaaaaattacgccccgccctgccactcatcgcagtactgttgtaattcatt

aagcattctgccgacatggaagccatcacagacggcatgatgaacctgaatcgccagcggcatcagcaccttgtcgccttg
cgtataatatttgcccatggtgaaaacgggggcgaagaagttgtccatattggccacgtttaaatcaaaactggtgaaactca
cccagggattggctgagacgaaaaacatattctcaataaaccctttagggaaataggccaggttttcaccgtaacacgccac
atcttgcgaatatatgtgtagaaactgccggaaatcgtcgtggtattcactccagagcgatgaaaacgtttcagtttgctcatg
gaaaacggtgtaacaagggtgaacactatcccatatcaccagctcaccgtctttcattgccatacg

pCSW3 (6379 bp):
tctagagctgagttggctgctgccaccgctgagcaataactagcataaccccttggggcctctaaacgggtcttgaggggtt
ttttgctgaaaggaggaactatatccgcgggatccagtctcgagagtagtactagagatctcggaagagagtcaattcagg
gtggtgaatgtgaaaccagtaacgttatacgatgtcgcagagtatgccggtgtctcttatcagaccgtttcccgcgtggtgaa
ccaggccagccacgtttctgcgaaaacgcgggaaaaagtggaagcggcgatggcggagctgaattacattcccaaccgc
gtggcacaacaactggcgggcaaacagtcgttgctgattggcgttgccacctccagtctggccctgcacgcgccgtcgca
aattgtcgcggcgattaaatctcgcgccgatcaactgggtgccagcgtggtggtgtcgatggtagaacgaagcggcgtcg
aagcctgtaaagcggcggtgcacaatcttctcgcgcaacgcgtcagtgggctgatcattaactatccgctggatgaccagg
atgccattgctgtggaagctgcctgcactaatgttccggcgttatttcttgatgtctctgaccagacacccatcaacagtattatt
ttctcccatgaagacggtacgcgactgggcgtggagcatctggtcgcattgggtcaccagcaaatcgcgctgttagcggg
cccattaagttctgtctcggcgcgtctgcgtctggctggctggcataaatatctcactcgcaatcaaattcagccgatagcgg
aacgggaaggcgactggagtgccatgtccggttttcaacaaaccatgcaaatgctgaatgagggcatcgttcccactgcga
tgctggttgccaacgatcagatggcgctgggcgcaatgcgcgccattaccgagtccgggctgcgcgttggtgcggatatct
cggtagtgggatacgacgataccgaagacagctcatgttatatcccgccgttaaccaccatcaaacaggattttcgcctgct
ggggcaaaccagcgtggaccgcttgctgcaactctctcagggccaggcggtgaagggcaatcagctgttgcccgtctca
ctggtgaaaagaaaaaccaccctggcgcccaatacgcaaaccgcctctccccgcgcgttggccgattcattaatgcagct
ggcacgacaggtttcccgactggaaagcgggcagtgagcgcaacgcaattaatgtgcgctgcagcccgtacctctagtag
agtcgagggggaacccggccgcgtccggcgcccccgccgccttcgacgagatcccgcaaaagcggcctttgactccct
gcaagcctcagcgaccgaatatatcggttatgcgtgggcgatggttgttgtcattgtcggcgcaactatcggtatcaagctgt
ttaagaaattcacctcgaaagcaagctgataaaccgatacaattaaaggctccttttggagccttttttttggagattttcaacg
tgaaaaaattattattcgcaattcctttagttgttcctttctattctcactccgctgaaactgttgaaagttgtttagcaaaacctcat
acagaaaattcatttactaacgtctggaaagacgacaaaactttagatcctctagctagatgtacatgccttgaccttgatgag
gcggcgtgagctacaatcaatactcgataattgtgagcggataacaattactagtatctgagttgaagaggtgacgtcatgaa
caaaggtgtaatgcgaccgggccatgtgcagctgcgtgtactggacatgagcaaggccctggaacactacgtcgagttgc
tgggcctgatcgagatggaccgtgacgaccagggccgtgtctatctgaaggcttggaccgaagtggataagttttccctgg
tgctacgcgaggctgacgagccgggcatggattttatgggtttcaaggttgtggatgaggatgctctccggcaactggagc
gggatctgatggcatatggctgtgccgttgagcagctacccgcaggtgaactgaacagttgtggccggcgcgtgcgcttcc
aggccccctccgggcatcacttcgagttgtatgcagacaaggaatatactggaaagtggggtttgaatgacgtcaatcccg
aggcatggccgcgcgatctgaaaggtatggcggctgtgcgtttcgaccacgccctcatgtatggcgacgaattgccggcg
acctatgacctgttcaccaaggtgctcggtttctatctggccgaacaggtgctggacgaaaatggcacgcgcgtcgcccag
tttctcagtctgtcgaccaaggcccacgacgtggccttcattcaccatccggaaaaaggccgcctccatcatgtgtccttcca
cctcgaaacctgggaagacttgcttcgcgccgccgacctgatctccatgaccgacacatctatcgatatcggcccaacccg
ccacggcctcactcacggcaagaccatctacttcttcgacccgtccggtaaccgcaacgaagtgttctgcgggggagatta
caactacccggaccacaaaccggtgacctggaccaccgaccagctgggcaaggcgatcttttaccacgaccgcattctca
acgaacgattcatgaccgtgctgacctgagaattctaagtagctgacaagagacaggatgaggatcgtttcgcatgattgaa
caagatggattgcacgcaggttctccggccgcttgggtggagaggctattcggctatgactgggcacaacagacaatcgg
ctgctctgatgccgccgtgttccggctgtcagcgcaggggcgcccggttctttttgtcaagaccgacctgtccggtgccctg
aatgaactgcaagacgaggcagcgcggctatcgtggctggccacgacgggcgttccttgcgcagctgtgctcgacgttgt

cactgaagcgggaagggactggctgctattgggcgaagtgccggggcaggatctcctgtcatctcaccttgctcctgccg
agaaagtatccatcatggctgatgcaatgcggcggctgcatacgcttgatccggctacctgcccattcgaccaccaagcga
aacatcgcatcgagcgagcacgtactcggatggaagccggtcttgtcgatcaggatgatctggacgaagagcatcaggg
gctcgcgccagccgaactgttcgccaggctcaaggcgcacatacccgacggcgaggatctcgtcgtgacccatggcgat
gcctgcttgccgaatatcatggtggaaaatggccgcttttctggattcatcgactgtggccggctgggtgtggcggaccgct
atcaggacatagcgttggctacccgtgatattgctgaagagcttggcggcgaatgggctgaccgcttcctcgtgctttacgg
tatcgccgctcccgattcgcagcgcatcgccttctatcgccttcttgacgagttcttctgagcgggactctggggttcgggtac
cgagctcgaattcgtaatcatgtcatagctgtttcctgtgtgaaattgttatccgctcacaattccacacaacatacgagccgg
aagcataaagtgtaaagcctggggtgcctaatgagtgagctaactcacattaattgcgttgcgctcactgcccgctttccagt
cgggaaacctgtcgtgccagctgcattaattcagatccttccgtatttagccagtatgttctctagtgtggttcgttgtttttgcgt
gagccatgagaacgaaccattgagatcatgcttactttgcatgtcactcaaaaattttgcctcaaaactggtgagctgaatttt
gcagttaaagcatcgtgtagtgtttttcttagtccgttacgtaggtaggaatctgatgtaatggttgttggtattttgtcaccattca
tttttatctggttgttctcaagttcggttacgagatccatttgtctatctagttcaacttggaaaatcaacgtatcagtcgggcggc
ctcgcttatcaaccaccaatttcatattgctgtaagtgtttaaatctttacttattggtttcaaaacccattggttaagccttttaaact
catggtagttattttcaagcattaacatgaacttaaattcatcaaggctaatctctatatttgccttgtgagttttcttttgtgttagttc
ttttaataaccactcataaatcctcatagagtatttgttttcaaaagacttaacatgttccagattatattttatgaattttttttaactgg
aaaagataaggcaatatctcttcactaaaaactaattctaatttttcgcttgagaacttggcatagtttgtccactggaaaatctc
aaagcctttaaccaaaggattcctgatttccacagttctcgtcatcagctctctggttgctttagctaatacaccataagcattttc
cctactgatgttcatcatctgagcgtattggttataagtgaacgataccgtccgttctttccttgtagggttttcaatcgtggggtt
gagtagtgccacacagcataaaattagcttggtttcatgctccgttaagtcatagcgactaatcgctagttcatttgctttgaaa
acaactaattcagacatccttcggaaaaagagttggtagctcttgatccggcaaacaaaccaccgctggtagcggtggtttttt
ttgtttgcaagcagcagattacgcgcagaaaaaaaggatctcaagaagatcctttgatcttttctacggggtctgacgctcagt
ggaacgaaaactcacgttaagggattttggtcatgagattatcaaaaaggatcttcacctagatccttttggttcatgtgcagct
ccatcagcaaaaggggatgataagtttatcaccaccgactatttgcaacagtgccgttgatcgtgctatgatcgactggatcg
gcggggcctggcggcgcctcgccgtcgcagaaccaggcggtggcgtacaccgtcgcctcggtcggcccgtagagattg
gcgatcccgaccgcagcaccaccgagaacgtccccgacgtggccgaccagcccgtcatcgtcaacgcctgaccgcggt
gcggacaggccgtgtcgcgaccggccgtgcggaattaagccggcccgtaccctgtgaatagaggtccgctgtgacacaa
gaatccctgttacttctcgaccgtattgattcggatgattcctacgcgagcctgcggaacgaccaggaattctgggagccgct
ggcccgccgagccctggaggagctcgggctgccggtgccgccggtgctgcgggtgcccggcgagagcaccaacccc
gtactggtcggcgagcccgacccggtgatcaagctgttcggcgagcactggtgcggtccggagagcctcgcgtcggagt
cggaggcgtacgcggtcctggcggacgccccggtgccggtgccccgcctcctcggccgcggcgagctgcggcccgg
caccggagcctggccgtggccctacctggtgatgagccggatgaccggcaccacctggcggtccgcgatggacggcac
gaccgaccggaacgcgctgctcgccctggcccgcgaactcggccgggtgctcggccggctgcacagggtgccgctga
ccgggaacaccgtgctcaccccccattccgaggtcttcccggaactgctgcgggaacgccgcgcggcgaccgtcgagg
accaccgcgggtggggctacctctcgccccggctgctggaccgcctggaggactggctgccggacgtggacacgctgc
tggccggccgcgaaccccggttcgtccacggcgacctgcacgggaccaacatcttcgtggacctggccgcgaccgagg
tcaccgggatcgtcgacttcaccgacgtctatgcgggagactcccgctacagcctggtgcaactgcatctcaacgccttcc
ggggcgaccgcgagatcctggccgcgctgctcgacggggcgcagtggaagcggaccgaggacttcgcccgcgaact
gctcgccttcaccttcctgcacgacttcgaggtgttcgaggagaccccgctggatctctccggcttcaccgatccggagga
actggcgcagttcctctgggggccgccggacaccgcccccggcgcctgacgccccgggccgcccggcgccgccccc
ggcccccggcggccgcccggagccccgcccgcgctcgggagccccgggcccgcgccgaagcccgctgctgcgagc
tagc

Reference:

1 Kieser, T., Bibb, M. J., Buttner, M. J., Chater, K. F., and Hopwood, D. A. *Practical Streptomyces Genetics*. (John Innes Foundation, Norwich, U.K., 2000).

2 Wang, W. *et al.* An engineered strong promoter for streptomycetes. *Appl. Environ. Microbiol.* **79**, 4484-4492 (2013).

3 Xu, G. *et al.* "Pseudo" gamma-butyrolactone receptors respond to antibiotic signals to coordinate antibiotic biosynthesis. *J. Biol. Chem.* **285**, 27440-27448 (2010).

4 Sun, J., Kelemen, G. H., Fernandez-Abalos, J. M. & Bibb, M. J. Green fluorescent protein as a reporter for spatial and temporal gene expression in *Streptomyces coelicolor* A3(2). *Microbiology* **145 (Pt 9)**, 2221-2227 (1999).

5 Wang, J. *et al.* A novel role of 'pseudo'gamma-butyrolactone receptors in controlling gamma-butyrolactone biosynthesis in *Streptomyces*. *Mol. Microbiol.* **82**, 236-250 (2011).
